# Functional and Genome Sequence-Driven Characterization of *tal* Effector Gene Repertoires Reveals Novel Variants With Altered Specificities in Closely Related Malian *Xanthomonas oryzae* pv. *oryzae* Strains

**DOI:** 10.3389/fmicb.2018.01657

**Published:** 2018-08-06

**Authors:** Hinda Doucouré, Alvaro L. Pérez-Quintero, Ganna Reshetnyak, Cheick Tekete, Florence Auguy, Emilie Thomas, Ralf Koebnik, Boris Szurek, Ousmane Koita, Valérie Verdier, Sébastien Cunnac

**Affiliations:** ^1^IRD, Cirad, Université de Montpellier, IPME, Montpellier, France; ^2^Laboratoire de Biologie Moléculaire Appliquée, Faculté des Sciences et Techniques, Université des Sciences Techniques et Technologiques de Bamako, Bamako, Mali

**Keywords:** rice, *Xanthomonas oryzae*, bacterial leaf blight, TAL effector, Mali, disease resistance

## Abstract

Rice bacterial leaf blight (BLB) is caused by *Xanthomonas oryzae* pv. *oryzae* (*Xoo*) which injects Transcription Activator-Like Effectors (TALEs) into the host cell to modulate the expression of target disease susceptibility genes. *Xoo* major-virulence TALEs universally target susceptibility genes of the SWEET sugar transporter family. TALE-unresponsive alleles of *OsSWEET* genes have been identified in the rice germplasm or created by genome editing and confer resistance to BLB. In recent years, BLB has become one of the major biotic constraints to rice cultivation in Mali. To inform the deployment of alternative sources of resistance in this country, rice lines carrying alleles of *OsSWEET14* unresponsive to either TalF (formerly Tal5) or TalC, two important TALEs previously identified in West African *Xoo*, were challenged with a panel of strains recently isolated in Mali and were found to remain susceptible to these isolates. The characterization of TALE repertoires revealed that *talF* and *talC* specific molecular markers were simultaneously present in all surveyed Malian strains, suggesting that the corresponding TALEs are broadly deployed by Malian *Xoo* to redundantly target the *OsSWEET14* gene promoter. Consistent with this, the capacity of most Malian *Xoo* to induce *OsSWEET14* was unaffected by either *talC*- or *talF*-unresponsive alleles of this gene. Long-read sequencing and assembly of eight Malian *Xoo* genomes confirmed the widespread occurrence of active TalF and TalC variants and provided a detailed insight into the diversity of TALE repertoires. All sequenced strains shared nine evolutionary related *tal* effector genes. Notably, a new TalF variant that is unable to induce *OsSWEET14* was identified. Furthermore, two distinct TalB variants were shown to have lost the ability to simultaneously induce two susceptibility genes as previously reported for the founding members of this group from strains MAI1 and BAI3. Yet, both new TalB variants retained the ability to induce one or the other of the two susceptibility genes. These results reveal molecular and functional differences in *tal* repertoires and will be important for the sustainable deployment of broad-spectrum and durable resistance to BLB in West Africa.

## Introduction

Genetic resistance is arguably the most sustainable strategy to control microbial diseases threatening crop production. However, effectiveness of resistance genes deployment in agricultural settings is contingent on a number of environmental and biological factors such as the spectrum of activity of the Resistance genes and the genetic diversity of pathogen populations, notably, with regards to the prevalence of resistance eliciting or suppressing factor(s) (Boyd et al., 2013; Brown, 2015).

Bacterial leaf blight (BLB) is a rice (*Oryza sativa*) foliar disease occurring in most rice growing regions. It has long been recognized in Asia as a serious yield limiting factors. BLB is considered as one of the three most important diseases of rice, causing yield reduction of 20–50% in extreme cases. *Xanthomonas oryzae* pv. *oryzae (Xoo)*, the gram negative bacteria responsible for BLB is a vascular pathogen that gains entry into plant tissues through hydathodes and wounds. *Xanthomonas oryzae* pv. *oryzicola (Xoc)* bacteria belong to another pathovar of the species and cause bacterial leaf streak (BLS) of rice, a disease less destructive than BLB but which is gaining in importance (Niño-Liu et al., 2006).

To successfully colonize its host and provoke significant disease symptoms, *Xoo* requires virulence proteins from the Transcription Activator-Like Effectors (TALEs) family. TALEs are injected into the host cell via the molecular syringe of the Type III Secretion System and subsequently localize to the nucleus where they molecularly mimic eukaryotic transcription factors and upregulate the expression of target genes. The central repeat region (CRR) of TALEs is responsible for recognition and binding to a specific target DNA sequence also termed effector binding element (EBE). The CRR domain is typically composed of 10–30 modular tandem repeats of 33–35 amino acids. The primary sequence of these repeats is highly conserved except at positions 12 and 13 which are referred to as repeat variable diresidue (RVD) (Boch and Bonas, 2010; Bogdanove et al., 2010). Structural insight into the features of TALE-DNA molecular complexes revealed that the CRR wraps around the DNA helix with the second residues of each RVDs interacting directly with a cognate nucleobase (Deng et al., 2012; Mak et al., 2012). The nature of each RVD determines affinity for a specific nucleotide in a linear fashion along the sequence of RVD in the TALE CRR and the target DNA sequence. The landmark elucidation of this TALE-DNA binding code (Boch et al., 2009; Moscou and Bogdanove, 2009) fostered the development of bioinformatic tools for the computational prediction of TALE target sequences (Moscou and Bogdanove, 2009; Doyle et al., 2012; Grau et al., 2013; Pérez-Quintero et al., 2013) and the design of artificial TALEs with tailored specificity (Boch et al., 2009; Morbitzer et al., 2010).

The functional interplay between TALEs and their rice gene targets is a major determinant of disease or resistance between *Xoo* strains and rice genotypes. When induction of a TALE target gene is demonstrated to make a positive contribution to disease outcome, this gene is termed a susceptibility gene. Documented BLB susceptibility host gene targets of TALEs include the transcription elongation factor *OsTFIIAγ1* and the b-ZIP transcription factor *OsTFX1* that were shown to be induced by TALE effectors from Philippine *Xoo* strains and have a mild effect on disease severity (White and Yang, 2009). In contrast, *OsSWEET* genes belonging to clade III of the family function as major susceptibility genes (Streubel et al., 2013). *SWEET* genes codes for membrane transporters with affinity for sugars and are primarily hypothesized to promote release of sucrose in the apoplast to provide a source of carbohydrate for bacterial multiplication (Bezrutczyk et al., 2018). *Xoo* strains do not monolithically target a single *OsSWEET* gene but rather have evolved TALEs inducing one of three *OsSWEET* clade III homologs: PthXo1 from the Philippine strain PXO99^A^ (Yang et al., 2006) targets Os*SWEET11* while PthXo2 from *Xoo* JXO1^A^ and MAFF311018 strains from Japan targets Os*SWEET13* (Zhou et al., 2015). Finally, in a remarkable example of convergent evolution, *OsSWEET14* stands out as being targeted by TALEs from geographically diverse and distantly related *Xoo* strains at the level of several distinct or overlapping EBEs in its promoter: AvrXa7 from strain PXO86 (Philippines) and PthXo3 from strain PXO61 (Philippines) (Antony et al., 2010) as well as Tal5 and TalC from African *Xoo* strains (Yu et al., 2011; Streubel et al., 2013). To date, the only African TALEs shown to target a clade III *OsSWEET* susceptibility gene are Tal5 from the Malian strain MAI1 and TalC from the Burkinabe strain BAI3. A *talC* mutant strain is unable to cause disease indicating that this effector is a major virulence TALE of the BAI3 strain (Yu et al., 2011; Streubel et al., 2013). To harmonize the nomenclature of African *Xoo* TALEs, Tal5 has been recently renamed TalF (Tran et al., 2018) and will be referred accordingly hereafter.

Resistance breeding is the only sustainable BLB control strategy in the field and more than 40 resistance loci have been characterized. With the notable exception of Pattern Recognition Receptors-encoding *Xa4*, *Xa21*, and *Xa23* genes, most BLB resistance systems described to date are based on the detection or the impairment of TALE activity (Zhang and Wang, 2013; Zuluaga et al., 2017) which further illustrates the critical status of this family of type III virulence effectors in the rice-*Xoo* evolutionary arms race. One type of host immunity relies on so-called ‘executor’ genes such as *Xa10*, *Xa23*, or *Xa27* that harbor a decoy TALE EBE in their promoter and act as triggers of a massive immune response upon infection attempts and promiscuous activation by a cognate TALE (Zhang et al., 2015).

Another recurring type of immunity originates from mutated alleles of gene promoters that confer a loss of TALE responsiveness to the corresponding *OsSWEET* susceptibility gene thereby hindering the establishment of proper bacterial growth conditions and preventing host tissues colonization (Hutin et al., 2015a). For example, the naturally occurring recessive resistance alleles *xa13* of *OsSWEET11* and *xa25* of *OsSWEET13* are, respectively, unresponsive to PthXo1 (Chu et al., 2006; Yang et al., 2006) and PthXo2 (Liu et al., 2011; Zhou et al., 2015) due to sequence polymorphism in the EBE recognized by the corresponding TALE. Recently, Hutin et al. (2015b) reported on *xa41(t)*, a resistance allele of *OsSWEET14* from the African wild rice species *O. barthii* that is also present in all examined cultivated varieties of the African *O. glaberrima* species. The *xa41(t)* promoter contains a 18 bp deletion spanning the AvrXa7 and TalF EBEs sequences and conferred resistance to half of the strains from a representative worldwide *Xoo* panel including six African strains from Burkina Faso, Niger, and Mali (Hutin et al., 2015b) which thus presumably rely solely on TalF for *OsSWEET14* activation.

Both the results of bioinformatic predictions of TALE target for strains with uncharacterized TALE repertoire (Pérez-Quintero et al., 2013; Grau et al., 2016; Quibod et al., 2016) and the consistent functional data on several *Xoo* TALE-*SWEET* pairs (Yang et al., 2006; Yu et al., 2011; Li et al., 2012; Zhou et al., 2015), support the view that this clade virtually act as universal BLB susceptibility genes. This and the existence of naturally occurring TALE-unresponsive *OsSWEET* resistance alleles in the rice germplasm hinted to a BLB resistance engineering strategy by genome editing of TALE EBEs in the promoter of *OsSWEET* genes. Pioneering work by Li et al. (2012) provided a proof of this concept by editing the AvrXa7 EBE upstream of *OsSWEET14* and conferring disease resistance to an Asian *Xoo* strain carrying this effector. A subsequent attempt to edit the TalF or the TalC EBE in the *OsSWEET14* promoter to create TALE-unresponsive resistance alleles tailored against African *Xoo* strains achieved immunity solely against those relying on TalF (Blanvillain-Baufumé et al., 2017). Intriguingly, the susceptibility of TalC-EBE edited lines to the TalC-relying strain BAI3 was unaffected even though none of the clade III *OsSWEET*, including *OsSWEET14*, was upregulated in these edited lines. This led to the conclusion that clade III *OsSWEET* induction is not an absolute requirement for BLB and that TalC also likely targets a genetically redundant susceptibility gene (Blanvillain-Baufumé et al., 2017).

In the past decade, rice has been recognized as a strategic crop and its cultivation has gained in importance in Africa. On this continent, BLB was first reported in Mali in 1979 and later found to occur in Senegal, Niger, Nigeria, Gabon, Mauritania, Benin, and Cameroon (Verdier et al., 2012). Probably due in part to surface extension and crop intensification, BLB is repeatedly observed in countries of the region, notably in Mali where rice pathologists have witnessed increased incidence and a marked susceptibility for local varieties in the field (Gonzalez et al., 2007; Sarra et al., 2010; Afolabi et al., 2015). Phylogenetic analysis of *Xoo* strains indicate that the African *Xoo* lineage is genetically distinct from the Asian one (Gonzalez et al., 2007; Poulin et al., 2015). It is noteworthy that among several distinguishing features, African *Xoo* strains harbor a reduced *tal* effector gene repertoire of nine elements (Gonzalez et al., 2007) compared to as much as 19 genes in Asian *Xoo* (Booher et al., 2015). Virulence profiling on nearly isogenic lines has clustered African strains isolated before 2007 into three races with Malian strains all belonging to race A3 which is incompatible on all lines of the IRBB panel, including the IR24 parental variety (Gonzalez et al., 2007). Recent work in our laboratories has expended our collection with ∼60 additional *Xoo* strains from Mali collected between 2009 and 2013. Virulence profiling on IRBB isogenic lines and Malian rice varieties as well as molecular typing indicated that these contemporary Malian *Xoo* isolates exhibit diversity both in terms of genetic content and virulence profiles as compared to strains isolated earlier. Many of these isolates define novel *Xoo* races and several of them are even able to overcome, at least partially, all tested sources of resistance (Tekete and Verdier, manuscript in preparation). Although, the TALE content of *Xoo* strains often underlies their pathogenicity, its variability among Malian strains remains unexplored. Until recently, our main insight into the nature of African *Xoo* TALEs came from the characterization of TalC and TalF. However, we used single molecule sequencing and functional assay to characterize the TALE repertoires of three African strains including MAI1 from Mali which redundantly activate *OsSWEET14* via both TalC and TalF (Tran et al., 2018). This work also identified TalB, a second major virulence TALE of African *Xoo* strains which remarkably targets two rice susceptibility genes, *OsTFX1* and *OsERF#123* (Tran et al., 2018).

Our objective is to provide farmers with broad BLB resistance to contemporary Malian *Xoo* strains. Recently described rice lines harboring either TalF- or TalC-unresponsive *OsSWEET14* promoter alleles were therefore challenged with Xoo but were found to be susceptible to all tested Malian isolates. To understand this lack of resistance, the *tal* gene repertoires of Malian strains were characterized. Active TalC appeared strictly conserved in Malian *Xoo* and, with one exception, consistently associated with an active version of the redundant TALE TalF. Comparative analysis of TALE repertoires additionally uncovered two variants of the TalB group that have lost the ability to induce one of the two documented targets of this group. Overall, Malian *Xoo* TALE groups members displayed an unexpected degree of variability raising the question of the functional significance of these differences in the interaction with rice.

## Results

### *OsSWEET14* Promoter Alleles Unresponsive to Single African TALEs Confer no Resistance to Malian *Xoo* Strains

Considering that TalF (previously Tal5) has been originally identified in a Malian strain (Streubel et al., 2013) and that an *O. barthii* accession containing the natural TalF-unresponsive allele *xa41(t)* is susceptible to strain MAI1 but resistant to three other Malian strains (CFBP1951, MAI9, and MAI14) (Hutin et al., 2015b), *xa41(t)* could be an effective resistance allele to control BLB in Mali. We therefore sought to evaluate its efficiency against a larger set of contemporary Malian strains composed in majority of isolates collected between 2009 and 2013. For this, CG14, a cultivated *O. glaberrima* variety that harbors a functional *xa41(t)* (Hutin et al., 2015b) and the Azucena variety of *O. sativa*, acting as a susceptible positive control, were inoculated with 44 Malian strains (including MAI1, MAI9, MAI14 as references) using the standard leaf tip clipping assay. For each strain, the length of BLB lesions were measured 14 days post inoculation on both varieties and plotted in **Supplementary Figure S1**. Similar to the BAI3 control strain which relies on TalC rather that TalF for *OsSWEET14* induction (Yu et al., 2011; Tran et al., 2018), a large fraction of the Malian strains appeared equally proficient at causing symptoms on CG14 and Azucena. Only seven strains, including the PXO86 control which relies on the AvrXa7 TALE that is unable to induce *SWEET14* in *xa41(t)* (Hutin et al., 2015b), caused significant disease lesions on Azucena but were markedly less virulent on CG14 (average lesion length below 5 cm). This was thus an indication that *xa41(t)* is not broadly efficient against contemporary isolates.

**FIGURE 1:**
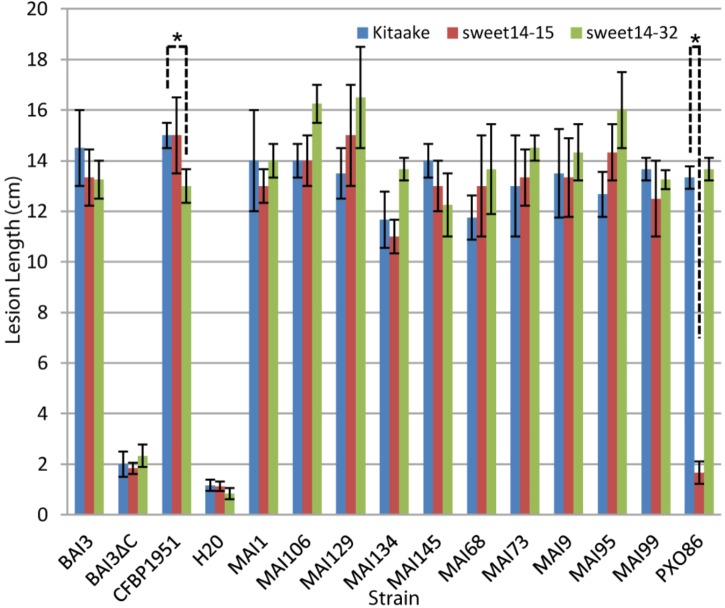
The TalF (Tal5) or TalC EBE edited lines do not exhibit measurable resistance against Malian *Xoo* strains. Barplot of mean lesion length measured on Kitaake wild type, *sweet14-32* or *sweet14-15* homozygous individuals for the edited alleles of the *OsSWEET14* promoter TalC and TalF (Tal5) EBE, respectively. Leaves of 6-week-old plants were inoculated using the leaf-clipping method. Lesion length was measured 2 weeks after inoculation. Error bars represent standard deviation calculated from 3 to 4 replicate measurements. The ‘BAI3ΔC’ strain corresponds to the BAI3 *talC^-^* knockout derivative. For each strain, Welch two sample *t*-tests were performed to test if average lesion length in wild-type Kitaake plants is superior to lesion length in one of the two mutant *sweet14* lines. Significant comparisons with *p*-value ≤ 0.05 are highlighted in the plot with an asterisk. This experiment was repeated four times with similar results.

Because this and previous experiments (Hutin et al., 2015b) with *xa41(t)*, could not use isogenic host rice backgrounds, interpretation on the causal role of *xa41(t)* on disease resistance can be confounded by other unrelated genetic factors. To unambiguously assess the contribution of a loss of TalF-responsiveness allele at *OsSWEET14* on resistance to Malian *Xoo*, we used the *OsSWEET14* promoter edited allele *sweet14-15*. It has been described previously and corresponds to a deletion of the entire AvrXa7 EBE and most (13 out of 19 bp) of the TalF EBE in a Kitaake cultivar (*O. sativa* ssp. *japonica*) parental background (Blanvillain-Baufumé et al., 2017). We also wanted to determine if a TalC-unresponsive *OsSWEET14* promoter edited allele could provide resistance to Malian *Xoo* and tested an homozygous line for the *sweet14-32* allele which has a large (16 out of 23 nt) deletion in the 3′ end of the TalC EBE (Blanvillain-Baufumé et al., 2017). Susceptibility assays of the edited lines and the parental Kitaake background were conducted with a restricted panel of Malian *Xoo* strains. As depicted in **Figure 1** and consistent with previous data, the BAI3 strain was equally virulent on the three rice genotypes. Similar to negative controls mock- or BAI3 *talC*- mutant-inoculated plants, the PXO86 control strain caused very short lesions, on *sweet14-15* plants as compared to wild type Kitaake. With the exception of CFBP1951 that caused slightly but significantly reduced lesions (*p*-value = 0.02473) on *sweet14-32* in this replicate of the experiment, Malian *Xoo* strains were similarly virulent on either of the *OsSWEET14* edited alleles than on the wild type control. We therefore conclude that none of the alleles conferred a strong resistance phenotype against any Malian *Xoo*. Altogether, these results demonstrate that not only TalC- but also TalF-unresponsive *OsSWEET14* alleles, either *xa41(t)* or *sweet14-15*, confer no or minor resistance against Malian *Xoo* strains. We further conclude that, in general, Malian strains do not rely solely on TalF for *SWEET* susceptibility gene induction.

### *tal* RFLP-Haplotypes of Malian *Xoo* Strains Show a Limited Diversity and Include Both *talC* and *talF*

**FIGURE 2:**
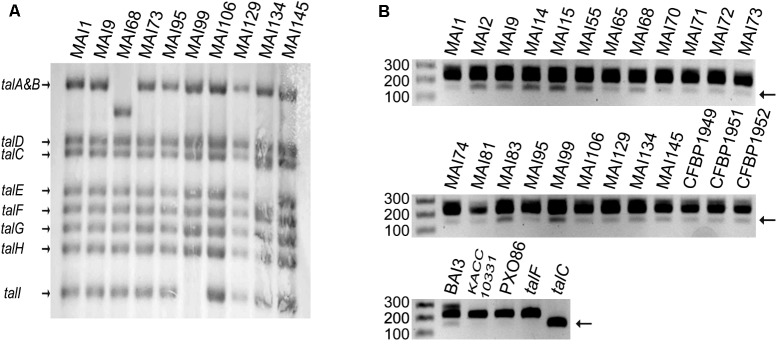
A Survey of *tal* gene diversity in a set of Malian *Xoo* strains. **(A)** Genomic patterns of *tal* content as determined by Southern blot. The genomic DNAs were digested with BamHI prior to electrophoresis on a 1% agarose gel. Following transfer, bands corresponding to *tal* sequences were detected with a probe encompassing part of the N-terminal coding region of TalF from MAI1. The names of *tal* genes corresponding to MAI1 fragments are indicated on the left. **(B)** Detection of *talC* in Malian *Xoo* genomes. A portion of the N-terminal region of *tal* coding sequences was amplified by PCR with primers flanking a segment that is specifically deleted in *talC* and separated on a 1.5% agarose gel. Detection of a shorter 152 bp product as compared to the predominant ∼224 bp product is indicative of the presence of *talC*. Purified plasmid DNA containing *talF* or *talC* and genomic DNA from strains KACC10331 (Korea) and PXO86 (Philippines) or BAI3 (Burkina Faso) were used, respectively, as negative or positive controls. The single gel image was broken down into three subpanels for assembling the figure. For each of them, the first lane was loaded with a 100 bp Marker and the numbers on the left indicate fragment size in bp. Strain names are indicated on top of the blot and gel images.

The conclusion that TalF is presumably not the only major TALE responsible for *SWEET* gene induction in Malian *Xoo* strains prompted us to explore the diversity of *tal* genes in our Malian strain collection. As a first step toward this goal, we conducted a preliminary screen of most of the Malian strains in our collection by Southern blotting with a PCR probe encompassing the 5′ part of the *talF* CDS on BamHI-digested genomic DNAs. As exemplified in **Figure 2A** on a restricted set of strains including those that were ultimately sequenced (see below), we detected a predominant haplotype of eight bands identical to the one obtained for our reference strain MAI1. Strain MAI68 defined a distinct haplotype differing at the level of the *talA–talB* band which migrated with a lower molecular weight. A third haplotype was also detected for strain MAI99 whose profile lacks the smallest *talI* band. Strain MAI134 defined a fourth haplotype with possibly an extra band beneath the *talC* one and the disappearance of the *talE* band. Importantly, the specific bands corresponding to *talC* and *talF* in strain MAI1 (Yu et al., 2011; Tran et al., 2018) were strictly conserved in all Malian strains examined, suggesting that these *tal* effector genes might be present in other Malian *Xoo* genomes as well.

In order to further ascertain the presence of a *talC* homolog in Malian strains, we designed a pair of PCR primers flanking a region in the 5′ portion of the coding sequence of *Xoo tal* genes that was found to be absent in the *talC* coding sequence (**Supplementary Figure S2**). As shown in **Figure 2B**, control PCRs with the BAI3 *talC* CDS cloned on a plasmid produced a band with a size consistent to the predicted 152 bp amplicon while using the cloned MAI1 *talF* CDS as a template yielded a band matching the size of the expected amplicon (224 bp). Additional control PCRs performed with the *talC*-containing strain BAI3 versus the Asian KACC10331 and PXO86 strains whose genomes are devoid of this gene revealed a ∼150 bp diagnostic band for the presence of *talC* in BAI3 only, thus verifying the specificity of this talC PCR marker. In the same experiment, we used it to also genotype a set of 24 genomic DNAs from Malian strains. Although the *talC* band had a weaker intensity, similar to PCR ran with BAI3 DNA as a template, we could repeatedly detect the *talC* diagnostic marker band for all tested Malian strains.

In conclusion, apart from three minor haplotypes observed only with single strains, Malian *tal* effector genes RFLP-profiles exhibit a low diversity with a major haplotype shared by most of the strains suggesting a limited divergence of TALE sequences across Malian *Xoo*. Furthermore, both *talF* and *talC* specific molecular markers were simultaneously detected in all surveyed Malian strains, suggesting that the corresponding TALEs are broadly deployed by Malian *Xoo* to redundantly target the *OsSWEET14* gene promoter.

### Malian *Xoo* Strains Exhibit *OsSWEET14*-Inducing Activity That Is Unaffected by Single TalC or TalF EBE Disruption

**FIGURE 3:**
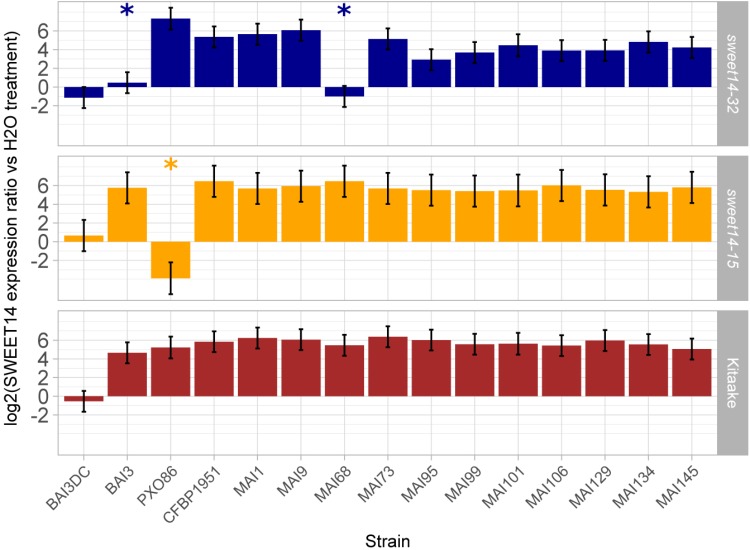
Profiling Malian strains *OsSWEET14*-induction activities on EBE edited rice lines. Q-RT-PCR was conducted on rice RNA extracted from leaf samples 48 h after infiltration of the designated bacterial strains (BAI3DC refers to the *talC* mutant of BAI3). The barplot reports least-square means and standard errors computed from a mixed linear model of the log_2_-transformed SWEET14 induction ratio relative to water treatment of the corresponding rice line (174 observations from four independent experiments). Likelihood ratio tests demonstrated that the model explaining *OsSWEET14* induction ratio as a function of rice line and strain and including a random component performed better than a model incorporating fixed effects alone (L-ratio = 195.48; *p*-value < 0.00001). The null hypothesis that induction ratio is inferior or equal to the value of the negative control strain BAI3DC was systematically rejected (*p*-value < 0.05 with Sidak adjustment method) except for treatments designated with an asterisk.

In order to functionally corroborate the hypothesis that a majority of the Malian *Xoo* strains deploys TalC and TalF to redundantly induce the *OsSWEET14* gene, we examined the ability of a set of 12 Malian strains, including those profiled above for *tal* effector genes, to induce *OsSWEET14* following leaf infiltration of the TalC EBE-edited line *sweet14-32*, the TalF EBE-edited line *sweet14-15* or the wild type Kitaake background variety. The resulting real time RT-PCR data is summarized in **Figure 3**. First, for all strains the *OsSWEET14* expression ratio relative to water infiltrations in Kitaake was superior to the negative control BAI3ΔtalC mutant strain at significant statistical levels (*p* < 0.05), indicating that these strains express at least a TALE targeting this gene. Second, with the exception of the control PX086 strain which is unable to induce *OsSWEET14* in a *sweet14-15* background because the AvrXa7 EBE of this allele is edited, all Malian strains caused *OsSWEET14* induction at ratios significantly superior to the corresponding control BAI3ΔtalC mutant strain revealing that they possess TalF EBE-independent *OsSWEET14* inducing activity. Finally, when assayed on the TalC EBE-edited line *sweet14-32*, and consistent with previous reports (Blanvillain-Baufumé et al., 2017), BAI3 failed to induce expression of *OsSWEET14* above the corresponding control BAI3ΔtalC mutant. Likewise, MAI68 did not induce *OsSWEET14* expression above the BAI3ΔtalC mutant control indicating that similar to BAI3 (Yu et al., 2011), this strain exclusively relies on TalC EBE-dependant activity for *OsSWEET14* targeting. Albeit to a more varying extent than on other rice backgrounds, all other Malian strains produced *OsSWEET14* expression ratio that were superior to the negative control BAI3ΔtalC mutant strain at significant statistical levels (*p* < 0.05), indicating that these strains possess TalC EBE-independent *Os*SWEET14 inducing activity.

This data therefore demonstrate that all 12 Malian strains possess *OsSWEET14*-induction activity and that, with the exception of MAI68, this activity is insensitive to single TalF- or TalC-EBE disruption. On another hand, our previous genotyping data advocates for the simultaneous presence of the *talF* and *talC* genes in these genomes. Taken together, these observations suggest that most Malian *Xoo* exhibit internal redundancy in TALE repertoires for *OsSWEET14* gene induction, and that they presumably rely simultaneously on TalF and TalC TALEs for that purpose.

### Whole Genome Sequencing of Eight Malian Strains

Single molecule, real-time (SMRT) sequencing (Pacific Biosciences) or ‘PacBio’ sequencing has been recently used for *X. oryzae* whole genome assembly and was shown to accurately and exhaustively reconstruct *tal* genomic sequences (Booher et al., 2015; Wilkins et al., 2015; Grau et al., 2016; Huguet-Tapia et al., 2016; Tran et al., 2018), surmounting the shortcomings of other NGS technologies to handle the repetitive nature of the CRR coding sequence. Available data on the genetic diversity of Malian *Xoo* strains is currently limited to MLVA typing (Poulin et al., 2015) and only one finished genome was sequenced (Tran et al., 2018). A more refined analysis of this genetic diversity, especially with regard to *tal* gene repertoires is critical to inform deployment of resistance genes and to design novel resistance by the creation of TALE unresponsive susceptibility genes alleles through EBE editing. We therefore performed *de novo* genome sequencing using the PacBio technology for selected Malian strains recently isolated (2010–2013) in the *Office du Niger* rice growing region with the aim of maximizing diversity in terms of virulence profile, genotype of the host of origin and *tal* haplotype. Hierarchical genome assembly of the PacBio data yielded a complete circular chromosomal sequence for all eight Malian strains with coverage above ∼130× (**Table 1**).

**Table 1:** Origin and genomic features of the Malian strains selected for genome sequencing.

Strain	Region	Site	Year	Host	Genome size (bp)	Coverage	GB accession
MAI68	Office du Niger	Niono	2010	Huang Huazhon	4703782	213	CP019085
MAI73	Office du Niger	Niono	2012	Adny11	4703982	373	CP019086
MAI95	Office du Niger	Niono	2012	Adny11	4705038	155	CP019087
MAI99	Office du Niger	Niono	2012	Adny11	4698819	193	CP019088
MAI106	Office du Niger	Niono	2012	Adny11	4705454	374	CP019089
MAI129	Office du Niger	Bewani 1	2013	Adny11	4703963	169	CP019090
MAI134	Office du Niger	Kala 3	2013	*O. longistaminata*	4730142	263	CP019091
MAI145	Office du Niger	Kouroumari	2013	Kogoni91-1	4703977	136	CP019092


**FIGURE 4:**
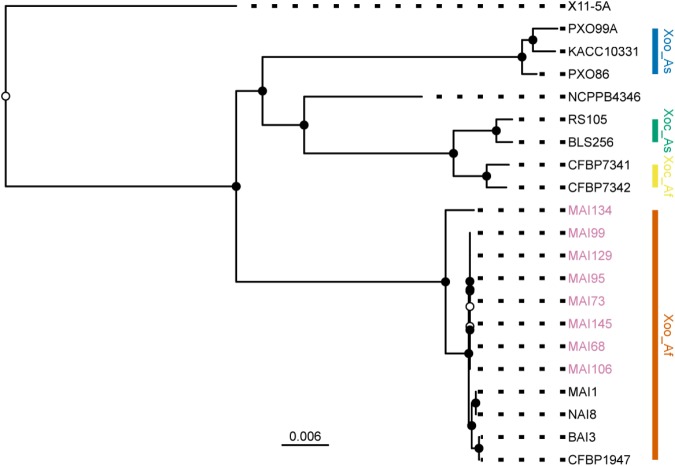
Position of the sequenced Malian *Xoo* strains in the *X. oryzae* phylogeny. Plot of the RAxML tree obtained from a core genome SNPs alignment composed of the PacBio sequenced Malian strain haplotypes (pink strain labels) and reference strains representative of major genetic groups in the *X. oryzae* phylogeny. The *X. oryzae* X11-5A strain served as an outgroup for rooting the tree. The main clades are highlighted with a colored vertical bar. Abbreviations for the label of the clades are as follows: Xoo, *X. oryzae* pv. *oryzae*; Xoc, *X. oryzae* pv. *oryzicola*; As, Asia; Af, Africa. Internal nodes were colored in black when bootstrap support values were above 80. The scale bar at the bottom of the tree reflects branch length in mean number of nucleotide substitutions per site.

Next, to examine the position of these strains in the established phylogeny of *X. oryzae*, a set of core genome SNPs was obtained using the parsnp module of the Harvest suite for genome multiple alignments (Treangen et al., 2014). On average, the core genome amounted to 67% of a genome sequence and a set of 129,898 SNPs could be called from this alignment. The analysis included genomes from reference strains representative of each of the previously defined major *X. oryzae* clades (Gonzalez et al., 2007; Triplett et al., 2011; Hajri et al., 2012): Asian *Xoo*, Asian *Xoc*, African *Xoc*, the *X. campestris* pv. *leersiae* NCPPB4346 strain, a pathogen of southern cutgrass (Parkinson et al., 2009; Triplett et al., 2011) and African *Xoo*. Finally, the X11-5A US strain, belonging to a more distant clade (Triplett et al., 2011), was used as an outgroup for rooting the tree. Evolutionary relations were reconstructed using a maximum likelihood approach and the resulting best tree plotted on **Figure 4**. Consistent with the disease symptoms caused on rice and their sampling location, all eight newly sequenced Malian strains clustered within the African *Xoo* group (**Figure 4**). Interestingly, MAI134, the only strain isolated from tissues sampled from the perennial grass *O. longistaminata* (**Table 1**) branches out earlier and is the most divergent African *Xoo* in this dataset. The other seven genomes fall in a single group separate from the MAI1 reference and appear to be highly related with a number of polymorphic positions in pairwise comparisons of core SNPs haplotypes between strains of this group ranging only from 1 to 22 (**Supplementary Figure S3**). Accordingly, multiple genome alignment of the African *Xoo* sequences performed with Mauve (Darling et al., 2010) revealed a high degree of shared synteny and, with the exception of CFBP1947, as pointed out before (Tran et al., 2018), no major structural rearrangement (**Supplementary Figure S4**).

In conclusion, genomic analysis of the finished genomes obtained from eight Malian isolates confirms that they genetically belong to the African *Xoo* group and that, with the exception of MAI134, they form a highly related group.

### Comparative Analysis of Malian Strains *tal* Effector Gene Repertoires

**FIGURE 5:**
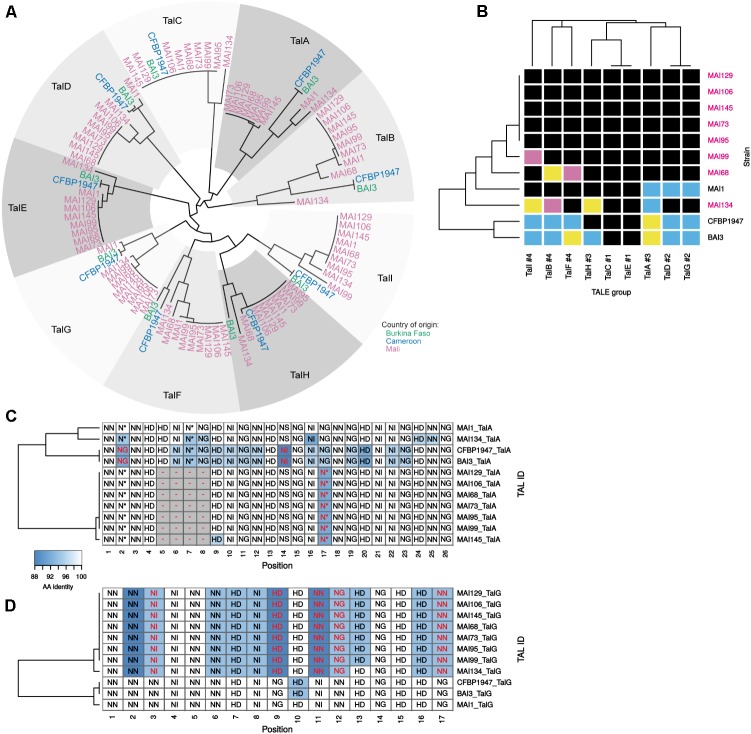
*In silico* analysis of the diversity of *tal* genes content of Malian strains. **(A)** DisTAL neighbor-joining phylogenetic tree and group classification of *tal* genes. All detected *tal* genes in available complete genomes of African strain (colored tip labels) were assigned to one of the previously defined African TALE groups (Tran et al., 2018) (gray level colored sectors) based on their position in the tree topology. **(B)** Diversity of African *Xoo* strains TALE RVD sequence variants in each TALE group. Within each TALE group (columns) and across strains (rows), individual cell colors code for distinct TALE RVD sequence variants. The number following the pound sign in the label of the TALE groups designates the total number of unique variants found for this group in this genome set. Malian strains whose genome was determined in this work are labeled in pink. Multiple alignments of the African TALE central repeat regions based on DisTAL repeat unit sequences for the TalA **(C)** and TalG **(D)** groups. Cell coloring reflects amino-acid identity between each repeat and the MAI1 TALE repeat at this position that was used as a reference. Each cell is labeled with the RVD encoded by the corresponding repeat. RVDs that are different from the corresponding MAI1 reference at this position are colored in red font. Gaps introduced to maximize the alignment score are colored in gray.

We then focused on the comparative analysis of *tal* effector gene repertoires to evaluate the nature and extent of TALE diversity in this set of genomes. To this end, the Malian genomes were searched for *tal* coding sequences (**Supplementary Table S1**). As before for strains MAI1, BAI3, and CFBP1947 (Tran et al., 2018), each was found to harbor nine *tal* genes that are highly syntenic (**Supplementary Figure S5**). These CDS were extracted and classified using the DisTAL module of the QueTAL suite that attempts to reconstruct evolutionary lineages based on the relatedness of the strings of unique repeat units amino acid sequences in the TALEs central region (Pérez-Quintero et al., 2015). The neighbor-joining tree obtained using DisTAL distances among African TALEs reproduced in **Figure 5A** indicates that TALEs from this new set of genomes belong to one of the nine African TALE groups previously defined by Tran et al. (2018). Furthermore, all genomes code for a single member of each group.

Thus, the newly sequenced Malian genomes do not reveal any unrelated TALE defining a new African group. However, the analysis of either RVD sequences (**Figure 5B**) or unique repeat units strings (‘DisTAL sequences’) (**Supplementary Figure S6**) similarity to measure intra-group diversity identified several new variants and yields some insight on *tal* repertoires variability across strains. As depicted in **Figure 5B**, when considering RVD sequences, new variants could be identified in our set of genomes in all but two TALE groups. For example, contemporary Malian strains encode a new variant of the TalD and TalG groups that were previously shown to be conserved across MAI1, BAI3, and CFBP1947 (Tran et al., 2018). Likewise, we found two new variants for the TalI and TalB groups. In contrast, despite a more comprehensive set of African genomes, the TalC and TalE groups remain strictly monomorphic at the RVD sequence level but polymorphic at the repeat sequences level (**Supplementary Figure S6**). Another observation, derived from the heatmap of **Figure 5B**, and mirroring the *tal* haplotypes shown in **Figure 2A**, is that strains MAI68, MAI99, and MAI134 exhibit distinct TALE RVD sequence variant profiles that are all different from the MAI1 reference. The five other strains displayed a Southern blot pattern identical to MAI1 (**Figure 2A**). Based on the analysis of their finished genome sequence, they share the same TALE RVD sequences content which is, however, distinct from MAI1 (**Figure 5B**).

**FIGURE 6:**
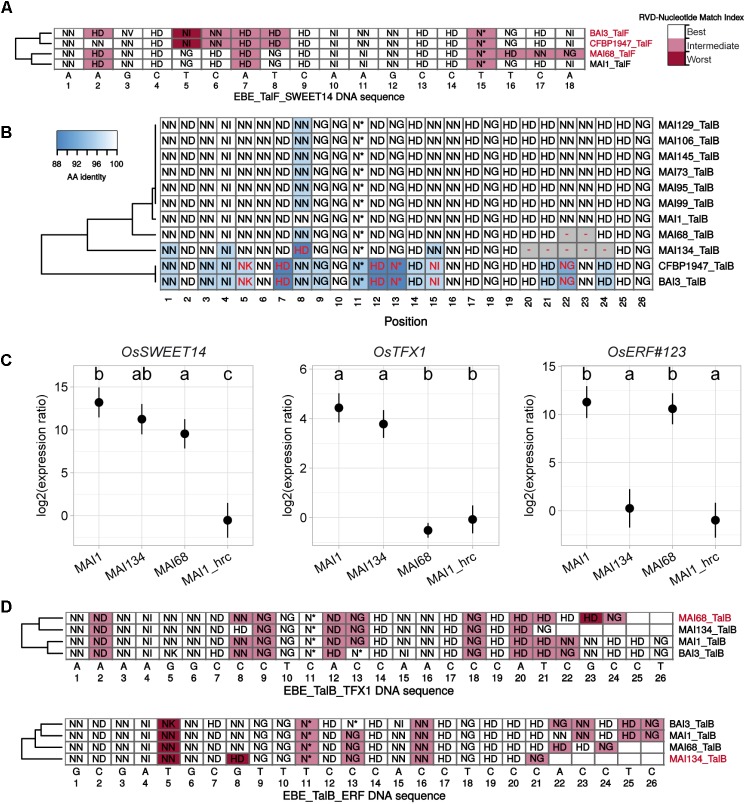
RVD sequence variability in the TalF and TalB groups has functional consequences on target gene induction activity. **(A)** Plain multiple alignment of selected TalF group RVD sequences (no gap allowed) along the DNA sequence (position| nucleotide as column labels) of TalF_MAI1_ predicted EBE on the promoter of *OsSWEET14* in the Nipponbare genome. A background color was assigned to each RVD based on the quality of the match between this RVD and the MAI1 TalF EBE nucleotide at this position. A RVD-nucleotide pair was classified in the ‘Best’ category if this nucleotide is the one with the best score in the RVD-nucleotide association matrix used by Talvez. It was assigned to the ‘Worst’ category if it corresponded to the worst score in the association matrix and in the ‘Intermediate’ category otherwise. TALE variants with labels colored in pink do not presumably recognize the corresponding EBE. **(B)** Multiple DisTAL repeat unit sequences alignments of the central repeat regions of TALEs from the TalB group. See the legend of **Figure 5** for details. **(C)** Target gene induction capacity of Malian strains MAI68 and MAI134 encoding TalB variants with repeat deletions. Q-RT-PCR was conducted on rice cultivar Kitaake total RNA extracted from leaf samples 48 h after infiltration of the designated bacterial strains. MAI1_hrc corresponds to the Type III Secretion System mutant derivative of MAI1. Average (points) and standard deviation (lines) of log_2_-transformed target gene (plot label) induction ratios relative to water treatment were computed from three biological replicate samples. Means having identical letters on top of the plotting area are not significantly different based on a Tukey’s HSD test (*a* = 0.05). This experiment was performed twice with similar results. **(D)** Plain multiple alignment of selected TalB group RVD sequences (no gap allowed) along the DNA sequence (position| nucleotide as column labels) of MAI1 TalF predicted EBE on the promoter of *OsTFX1* or *OsERF#123* in the Nipponbare genome. See the legend of **(A)** for details.

In order to examine the differences in repeat array regions underlying each variant within individual TALE groups, we generated in **Supplementary Figure S7** multiple alignments of DisTAL sequences that allowed the insertion of gaps to maximize alignments (Pérez-Quintero, 2017). Two main type of variation seems to occur in these alignments. The TalA group for example (**Figure 5C**) but also the TalB (see below and **Figure 6B**), TalH and TalI groups (**Supplementary Figure S7**) harbor variants that may have arisen from the insertion/duplication or deletion of an internal block of several contiguous repeats in an ancestor variant. A second type of variation, that could be described as single repeat polymorphism, occurs pervasively in most African TALE groups but one of the most extreme examples is probably the TalG group (**Figure 5D**) where up to 12 positions out of 17 contain 2–3 unique repeat types that may (positions 3, 9, 11, 12, and 17), or may not, hold polymorphic RVDs. This repeat polymorphism splits TalG variants into two subgroups: one of them is composed of variants from all contemporary Malian strains while the other contains variants from the previously sequenced African *Xoo* genomes. Regardless of the type of variation, within an African TALE group, variants differences are often predicted to have marked repercussions on their respective set of rice target genes (**Supplementary Figure S8**). Indeed, for example, none of predicted rice gene targets for the TalG variant of contemporary Malian strains is shared with the ones predicted for the other variant.

In conclusion, within established evolutionary TALE groups, genome sequencing and comparative analysis identified several novel African TALE variants with distinct predicted target specificities. Two of them, namely TalC and TalE, were however, found to be invariant in terms of RVD sequence.

### RVD Sequence Polymorphism in the TalF and TalB Group Is Associated With Distinct Induction Patterns of the Corresponding Rice Target Genes

To explain *tal* typing and *OsSWEET14*-induction profile data, we proposed above that, similar to MAI1 (Tran et al., 2018), a majority of the Malian *Xoo* strains possess a version of both TalF and TalC, each being active on *OsSWEET14*. Our Malian *Xoo* genome sequences conclusively support this hypothesis: all TalC variants are strictly identical in terms of RVD sequence and, apart from MAI68, all Malian TalF variant RVD sequences are also identical (**Figure 5B** and **Supplementary Figure S7**). TalF_MAI68_ appears to deviate from other members of the group only at the level of its last three repeats (**Supplementary Figure S7**). TalF_MAI68_ may fail to induce *OsSWEET14* because it is unable to recognize its EBE. Consistent with this view, while *OsSWEET14* is the fifth best predicted target of TalF_MAI1,_ it only ranks at the 3906th position in Talvez EBE predictions (Pérez-Quintero et al., 2013) for TalF_MAI68_ (see **Supplementary Figure S9**). In line with this, we examined the extent to which individual RVDs of active (MAI1) or inactive (BAI3) TalF variants are likely to recognize their cognate nucleotide along the DNA sequence of the TalF EBE in the *OsSWEET14* promoter (**Figure 6A**). TalF_BAI3_ fail to recognize this target sequence possibly due to suboptimal pairing between positions 5 and 8 (Tran et al., 2018). Each of the last three polymorphic RVD (position 15–18) of TalF_MAI68_, including the NN at position 17 which is present in place of the strong HD of TalF_MAI1_, are predicted to be suboptimal for recognition of the cognate nucleotide, leaving a productive recognition scaffold of only 14 RVDs. Altogether, this suggests that MAI68 lacks TalF activity because the corresponding TALE variant is unable to bind the canonical EBE in the *OsSWEET14* promoter of the Nipponbare genome.

It has been recently discovered that the MAI1 and BAI3 variants of TalB are both able to simultaneously upregulate two rice susceptibility genes, *OsTFX1* and *OsERF#123* (Tran et al., 2018). Our genome sequences analysis reveals, however, that two strains, MAI68 and MAI134 encode shorter TalB group variants with a deletion of a block of, respectively, 2 and 5 repeats, in the C-terminal portion of the central domain (**Figure 6B** and **Supplementary Figure S7**). This prompted us to examine this additional TALE group with documented targets for altered induction activity of polymorphic variants. To this end, strains MAI68, MAI134, and MAI1, acting as a positive induction control, were infiltrated into the leaf apoplast of Kitaake plant and tissues were sampled after 48 h. As shown in **Figure 6C**, induction of *OsTFX1*, *OsERF#123*, and *OsSWEET14*, as a control, was measured by qRT-PCR in total RNA extracted from theses samples. Consistent with data from **Figure 3**, all three strains induced *OsSWEET14* well above the background levels measured for the MAI1 Type III secretion minus mutant strain negative control. In contrast, *OsTFX1* and *OsERF#123* induction patterns proved to be different from *OsSWEET14*: while both genes were induced by the MAI1 positive control relative to the background levels defined by the MAI1 Type III secretion minus mutant values, MAI134 induced *OsTFX1* but not *OsERF#123* expression. The MAI68 strain had an opposite activity, inducing only *OsERF#123* but not *OsTFX1* expression (**Figure 6C**). This is a strong indication that these TalB variants have differentially lost the ability to recognize one of the documented targets of the group. We wondered if the inability to induce one of the two TalB susceptibility targets had an impact on the virulence of these strains on Kitaake. A Dunnet’s test comparing mean lesion length obtained on Kitaake with MAI68 or MAI134 against the value obtained with MAI1 found no statistical difference (Adjusted *p*-values > 0.05) in data from three out of four of the replicate experiments performed for **Figure 1**. It therefore appears that in our experimental conditions, those strains have no reproducible virulence defect and behave as MAI1.

To understand why, in contrast to TalB_MAI1_, TalB_MAI68_, and TalB_MAI134_ specifically fail to recognize the *OsTFX1* and *OsERF#123* EBEs, respectively, we first examined the results of Talvez predictions for these TALE-target pairs (**Supplementary Figure S9**). Consistent with target genes induction patterns, *OsTFX1* was absent from the list of the first 5000 best predictions of TalB_MAI68_, likewise for *OsERF#123* with TalB_MAI134_. Conversely, predictions scores for TalB_MAI68_ on *OsTFX1* and TalB_MAI134_ on *OsERF#123* where higher than the predictions scores for TalB_MAI1_ on the corresponding targets. As above, we also inspected the expected fitness of individual RVD-nucleotide pairs alongside target DNA sequences (**Figure 6D**). For the *OsTFX1* EBE, TalB_MAI68_ is shorter than active variants (24 versus 26 RVDs) and four of its last five RVD are suboptimal for recognition of the target nucleotides. Although TalB_MAI134_ is even shorter (21 versus 26 RVDs), there is no such stretch of RVD-nucleotide mismatches and the substitution of NN at position 8 for a strong HD may compensate for a potential decrease in affinity of a shorter version. At the *OsERF#123* EBEs, it is possible that this substitution may in the case of the short TalB_MAI134_ variant create a mismatch that disproportionately penalizes affinity for this DNA sequence.

In summary, comparative analysis of *tal* gene sequences assembled with the PacBio data identified variants in the TalF and TalB groups with modified rice gene target induction specificity relative to group founders in MAI1 and BAI3 strains. Intriguingly, the TalB group variants presumably remain functional but have lost the ability to induce one of the two documented susceptibility targets of TalB group founders without a detectable effect on virulence.

## Discussion

Bacterial leaf blight can represent a significant constraint to production in some rice growing areas of West Africa, notably in the irrigated perimeters of *Office du Niger* in Mali. A few resistance genes originally identified using Asian *Xoo* strains (Zhang and Wang, 2013) are also effective against West African strains (Gonzalez et al., 2007; Sarra et al., 2010) and could be readily deployed in breeding programs. To further expand the host genetic arsenal available to control BLB in Mali, this study initially aimed at assessing recently documented resistance alleles of the *OsSWEET14* susceptibility gene that loss responsiveness to the African TALE TalF for protection against Malian *Xoo* strains. Although *xa41(t)*, a *OsSWEET14* resistance allele identified in *O. barthii* and *O. glaberrima* African rice was previously shown to be effective against half of a worldwide diversity set of *Xoo* strains in a water-soaking assay after leaf infiltration (Hutin et al., 2015b), we unexpectedly found that most of the Malian strains isolated after 2009 were virulent on the CG14 accession bearing *xa41(t)* in leaf clipping assays (**Supplementary Figure S1**). In parallel, we tested the equivalent *sweet14-15* edited allele (Blanvillain-Baufumé et al., 2017) carrying also a lesion in the TalF EBE. Eleven Malian strains, including CFBP1951 and MAI9 that were previously shown to be controlled by the *xa41(t)* allele, were all as virulent on the *sweet14-15* genotype as on the parental Kitaake variety (**Figure 1**). In agreement with this, TalF is not the only TALE required for susceptibility gene induction by Malian strains because all Malian strains tested were able to up-regulate *OsSWEET14* in the TalF EBE edited line (**Figure 3**). We therefore propose that the resistance observed on rice accessions carrying *xa41(t)* against Malian strains is attributable to other unrelated resistance loci present in these genetic backgrounds. Furthermore, based on our data, TalF-unresponsive *OsSWEET14* alleles appear of limited practical value to control BLB caused by contemporary *Xoo* strains in Mali.

The lack of protective effect of TalF-unresponsive alleles is most probably due to the widespread if not the universal presence of an active *talC* gene in the genomes of contemporary Malian strains in addition to *talF*. Tran et al. (2018) recently discovered that the Malian *Xoo* strain MAI1 contains one active copy of both *talC* and *talF*. We further demonstrate that single EBE disrupted alleles of the *OsSWEET14* promoter still respond to MAI1 and to most of the 12 Malian *Xoo* tested (**Figure 3**). Moreover, *tal* haplotype characterization, PCR-based detection of *talC* sequences (**Figure 2**) and ultimately PacBio sequencing of eight *Xoo* strains (**Figure 5**) support the view that the Malian *Xoo* genetic diversity possess redundant TalF and TalC activities on *OsSWEET14*. Redundant targeting of the *SWEET* component of susceptibility is not unique to Malian *Xoo*. A significant proportion of tested Asian strains (∼40%) also adopted this strategy but implement it by targeting several (up to three) clade-III *OsSWEET* genes using a distinct TALE for each one of them (Parkinson et al., 2009). In principle, engineering TALE-unresponsive alleles for resistance against most Malian *Xoo* would require to edit both the TalC and TalF EBEs on the *OsSWEET14* promoter to abrogate induction of this susceptibility gene. Based on the phenotype of TalC EBE edited lines that remain susceptible to the Burkinabe BAI3 (Blanvillain-Baufumé et al., 2017) and the Malian MAI68 (**Figure 1**) strains possessing TalC-activity only, one would, however, predict that a double edited *OsSWEET14* promoter unresponsive to Malian strains will not provide resistance. The failure to confer enhanced BLB resistance against the BAI3 strain despite unchanged clade-III *OsSWEET* expression has been hypothesized to originate from the presence of another genetically redundant TalC target of unknown nature (Blanvillain-Baufumé et al., 2017). Our results provide evidence that TalC is both widespread and highly conserved in Malian *Xoo*. It will therefore be critical to identify this redundant target if broad resistance against the African *Xoo* characterized to date is to be engineered utilizing genome editing technologies.

PacBio sequencing, assembly and analysis of eight finished *Xoo* Malian genomes provided valuable insight on the genetic diversity of contemporary *Xoo* strains in this country. The phylogenetic position of some of the contemporary Malian strains, namely, MAI73, MAI95, MAI99, and MAI106 was previously investigated using a MLVA scheme (Poulin et al., 2015). They were all shown to belong to a sub-cluster composed of Malian strains collected between 2010 and 2012 in *Office du Niger* and separated from another sub-cluster including MAI1 and composed of Malian strains isolated earlier. Our phylogenetic tree based on whole genome core SNP markers (**Figure 4**) also displays such a dichotomy between MAI1 and more recently collected strains which form a group of highly related genomes both in terms of DNA sequence identity and overall genome collinearity. Interestingly, *Xoo* MAI134 is the most divergent genome belonging to the African *Xoo* group and it clusters separately from the other Malian strains. To our knowledge, it is the first *Xoo* genome of a strain isolated from a wild rice species, in that case, *O. longistaminata*. The apparent divergence relative to other Malian genomes could be a consequence of a separate evolutionary path for specialization on this wild host. Sequencing the genome of additional *Xoo* strains isolated from wild rice would help address this question.

Our analysis of the finished Malian *Xoo* genomes extends previous foundational work on three African *Xoo* genomes from strains isolated in Cameroon, Burkina Faso, and Mali (Huguet-Tapia et al., 2016; Tran et al., 2018). We further characterized the nature and extent of the diversity of the genomic repertoires of *tal* effector gene homologs in a larger dataset. One interesting finding is that the new genomes also individually encodes nine TALEs each belonging to one of the nine previously defined groups of evolutionary related TALEs. Similar approaches identified in the order of 30 such groups or classes in Asian *Xoo* (Grau et al., 2016; Quibod et al., 2016). The limited number of TALE groups in African *Xoo* is intriguing. It could be due to incomplete sampling of this diversity but also probably reflects a narrower genetic basis of African *Xoo* populations mirroring the limited diversity of traditional African rice host genotypes (Vaughan et al., 2008). This restricted number of TALE groups may be somehow counter balanced by intra-group variability. Indeed, we describe new variants for all of the variable TALE groups and the TalD and TalG groups that were previously thought to be conserved in terms of RVD sequences (Tran et al., 2018) appear to be variable as well. The observed differences within TALE groups encompass repeat amino acid sequence polymorphism outside the RVD, polymorphic RVDs and insertion/deletion events of stretches of repeat units. As the number of *tal* sequences in databases is dramatically increasing the molecular mechanisms responsible for *tal* sequence variation are beginning to be deciphered and seem to include both point mutation and recombination between repeats (Erkes et al., 2017; Pérez-Quintero, 2017; Tran et al., 2018).

Long-read sequencing has made TALE diversity mining a straightforward task. As this diversity in *Xoo* populations is being increasingly recognized, new fundamental issues about TALEs biology and evolution are emerging. These questions will also be relevant for rice breeding because they may inform genetic disease resistance design and deployment strategies. In this regard, one of the most important challenges is to understand the significance of RVD sequence variability or conservation within TALE groups in terms of the underlying selective forces promoting this diversity. Does variability entails functional differences in host target gene sets providing a fitness benefit? Or, is this variability simply neutral with respect to susceptibility target specificity because of the tolerance to mismatches of the RVD-nucleotide recognition code? Variability within a TALE group could be a sign that diversifying selection is driving evolution of the corresponding genes. Two selective forces may promote diversification of TALE: escaping detection by a decoy *R* gene while potentially maintaining control over the cognate susceptibility gene target(s) or overcoming loss-of-TALE-responsiveness resistance alleles of susceptibility genes. While strict conservation of some TALE groups, such as TalC and TalE (**Figure 5B**), is generally understood as a clue that cognate host gene targets are important for susceptibility, because of strong purifying selection, it is also perhaps the sign that these variants are not engaged by TALE-dependant immune systems in the genetic pool of African *Xoo* hosts.

The case of the TalF group in African *Xoo* illustrates some of these considerations. TalF_BAI3_ has previously been shown to be unable to recognize the *OsSWEET14* promoter in contrast to TalF_MAI1_ (Tran et al., 2018). We identified a novel TalF variant from strain MAI68 that is also presumably unable to up-regulate this susceptibility gene. To integrate this observation with our current knowledge, we propose an evolutionary scenario whereby ancestral Malian strains were equipped with a functional TalF_MAI1_-like TALE for *OsSWEET14* induction. However, the emergence of TALE-unresponsive *xa41(t)* resistance allele, which is present in all surveyed accessions of the domesticated *O. glaberrima* African species (Hutin et al., 2015b) must have exerted a strong selection pressure. Acquisition of *talC* lifted this barrier to colonization and resulted in the general spread of this effector gene in West African *Xoo* populations.

In this study, we also describe another case of target specificity shift for variants within the TalB group. The MAI1 and BAI3 founding members regulate two rice susceptibility genes, *OsTFX1* and *OsERF#123* (Tran et al., 2018). We discovered that strains MAI68 and MAI134 possess TalB variants with deletions of several repeats in the C-terminal region of the CRR and that they are incapable of inducing one of the two targets: *OsTFX1* for TalB_MAI68_ and *OsERF#123* for TalB_MAI134_. Considering that these strains did not show reduced virulence relative to MAI1 in our assays (**Figure 1**), it is possible that these susceptibility targets are genetically redundant. Alternatively, unknown mechanisms may compensate the absence of induction of one of the susceptibility genes.

Using the RVD-nucleotide association code, we attempted to mechanistically explain the failure of some TalF and TalB variants to recognize cognate EBEs (**Figures 6A,D**). While formal demonstration of these hypotheses will require further experimental evidence, they are consistent with the concept of ‘strong RVD’ (Streubel et al., 2012) and with the influence of CRR repeat number on the effect of RVD-nucleotide mismatches on overall DNA affinity (Rinaldi et al., 2017). Ours results argues in favor of the idea that RVD sequence variability within TALE groups is not always neutral relative to the set of direct host gene target(s) and leads to distinct targets induction patterns by RVD polymorphic TALEs. However, at least for the TalB group, there are substantial differences between the BAI3 and the major Malian variant (**Figure 6B**) with, as yet, no detectable consequence. We observed that prediction scores for the TalB and TalF variants that have lost targeting specificity for a host gene are consistently lower than scores for the variants that are able to recognize that target. Examining the overlap of predicted target sets between variants revealed that for many of the variable African *Xoo* TALE groups predicted specificity also varies greatly (**Supplementary Figure S8**), indicating that if any, relevant targets may not be consistently induced by variants. Ultimately, systematic approaches to study the effect of TALE variants on functionally relevant target gene induction should yield crucial insight on these issues. Wilkins et al. (2015) have observed a conservation of the specificity for confirmed target(s) of a reference *Xoc* TALE when variants within that group differed by no more than six RVDs. Reminiscent of our findings regarding African *Xoo* TalB variants, another study reported on several *Xoc* TALE groups where different variants may have contrasted abilities to induce predicted targets (Erkes et al., 2017). In the future, relational databases such as daTALbase (Pérez-Quintero et al., 2017), that integrate massive TALE-centered ‘omic’ information should facilitate even more comprehensive inquiries, and help explore for example whether ‘divergent’ variants of a TALE group are predicted to gain the capacity to induce a polymorphic version of the target in another rice genome.

## Conclusion

This work demonstrated that Malian *Xoo* populations circumvent unresponsive alleles of the susceptibility gene *OsSWEET14* by combining redundant *talF* and *talC* genes which makes this type of resistance unsuitable for control of BLB in the country. Genome sequence analysis further showed that most contemporary Malian strains are highly related but nevertheless harbor *tal* effector gene repertoires encoding polymorphic TALE groups that have contrasted abilities to induce documented susceptibility target genes, potentially underlying host adaptation at a small evolutionary scale.

## Materials and Methods

### Bacterial Strains, Growth Conditions and DNA Isolation

The *Xoo* bacterial strains used in this study for leaf clipping and infiltration assays were as follows: wild-type PXO86 (Vera Cruz, 1989), BAI3 (Gonzalez et al., 2007), the BAI3 *talC^-^* knockout derivative (Yu et al., 2011), a MAI1 Type III Secretion System mutant and Malian *Xoo* strains (Poulin et al., 2015; Tekete and Verdier, manuscript in preparation). The isolates were maintained in 15% glycerol at -80°C. All *Xoo* strains were cultivated on PSA (10 g/liter peptone, 10 g/liter sucrose, 1 g/liter glutamic acid, 15 g/liter Bacto Agar) except BAI3ΔTalC that was grown on PSA supplemented with 50 μg/ml kanamycin. The isolates were incubated at 28°C for 48 h.

For DNA extraction of Malian *Xoo*, two loops of bacterial cultures grown on PSA were washed twice with sterilized water. The genomic DNA was extracted using either the Wizard genomic DNA purification kit (Promega) for Southern Blot assays, or with the DNeasy DNA extraction kit (Qiagen) for Pacific Biosciences single molecule real time sequencing following the manufacturers’ instructions.

### Leaf Clipping Assay

To evaluate the virulence of Malian *Xoo* strains, three lines of rice were used: Kitaake wild-type, *sweet14-32* (the TalC EBE edited line) and *sweet14-15* (the TalF and AvrXa7 EBEs edited line) described in Blanvillain-Baufumé et al. (2017). Plants were grown under greenhouse conditions under the following cycles of 12 h of light at 28°C and 80% relative humidity and 12 h of dark at 25°C and 70% RH. For inoculation, 2 days old *Xoo* cultures were resuspended in sterilized water and leaves of 6-week-old plants were cut with scissors previously dipped in bacterial suspensions at an OD600 = 0.2. The infected plants were kept in the green house for 14 days until disease symptoms were evaluated by measuring lesion length on leaves.

### RNA Isolation and qRT-PCR

To assess the induction of the *OsSWEET14* gene, leaves of 3-week-old plants were infiltrated with a needleless syringe with sterilized water or bacterial suspensions at an OD600 adjusted to 0.5 in water. Segments of inoculated leaf were collected 48 h after inoculation. Samples were ground into powder using the Qiagen Tissue-Lyser system. Total RNA was extracted using Trizol reagent (Invitrogen) following the manufacturer’s instructions. After TURBO DNase treatment (Ambion), 1 μg RNA was reverse transcribed into cDNA using SuperScript III (Invitrogen). Real-time PCR was carried on a Lightcycler 480 System (Roche) with primer pairs specific for *OsSWEET14*, *OsTFX1*, *OsERF#123* or *EF-1 alpha* (GenBank accession: GQ848072.1) as described before (Blanvillain-Baufumé et al., 2017; Tran et al., 2018). Gene expression was calculated using the 2^-ΔΔCt^ method with *EF-1 alpha* acting as a reference gene and the data from the water inoculated leaves of the corresponding rice genotype as the reference condition.

### Statistical Analysis

Statistical tests for mean comparisons were performed in R (R Core Team, 2010) using standard functions. The R package nlme (Pinheiro et al., 2017) was used to perform linear mixed-effects modeling of *OsSWEET14* expression with log_2_-transformed qRT-PCR values computed as the response variable. This transformation was necessary to ensure homogeneity. The dataset included one value for each ‘strain’ and ‘rice line’ factor combinations obtained from four independent replicates of the whole experiment. The fixed effects of the final model included the variables ‘strain’ and ‘rice line’ and their interaction term. The structure of the random component consisted of a random intercept and a random slope for the ‘rice line’ factor both conditioned on a ‘replicate experiment ID’ factor. The procedure for model selection and validation followed advices from Zuur et al. (2009). Visual inspection of residual plots did not reveal any major deviation from homoscedasticity or normality. For *post hoc* analysis on the linear mixed effect model, pairwise comparisons were performed on least-squares means computed using the R package lsmeans (Lenth, 2016).

### Southern Blot Analysis

Genomic DNA of the Malian *Xoo* strains was digested by BamHI (New England Biolabs). The digested DNA was separated in 1% agarose gel at 50 Volt for 72 h at 4°C and transferred to a nylon membrane (Roche) overnight. A 560 bp fragment corresponding to the coding sequence of the N-terminal of TalF from MAI1 was used as a probe and PCR amplified using GCAGCTTCAGCGATCTGCTC and TCAGGGGGGCACCCGTCAGT primers. DIG-High prime DNA labeling and detection starter kit II (Roche) was applied for probe labeling, hybridization and detection procedures according to the manufacturer’s instructions.

### *talC* PCR Marker

To detect the presence of *talC* sequences in *Xoo*, a pair of primers (forward TCTGCGTGCAGCCGATGACCC and reverse CCACCAGTGCCTCGTGGTGCTG) was designed to anneal on sites flanking the deleted region (**Supplementary Figure S2**) and amplified a ∼152 bp fragment from *talC* and a ∼224 bp from typical *tal* sequences. PCR used the GoTaq DNA Polymerase (Promega) and thermal conditions were as follows: an initial denaturation at 95°C for 5 min, followed by 28 cycles of denaturation at 95°C for 30 s, annealing at 64°C for 30 s and extension at 72°C for 1 min, followed by a final extension at 72°C for 5 min. PCR amplicons were separated in 1.5% agarose gel at 100 V for 45 min.

### Genome Sequencing and Assembly

The *X. oryzae* pv. *oryzae* genome sequences were obtained using the SMRT technology (Eid et al., 2009). 20 kb SMRTbell templates libraries were sequenced on a PacBio RS II instrument with the P6-C4 chemistry at the Icahn Institute for Genomics and Multiscale Biology (New York, NY, United States). For each strain, 1–2 SMRT cells were employed to generate sequencing data equivalent to more than 100× genome coverage. Genome assembly was performed with version 3 of the HGAP pipeline (Rank et al., 2013) using default parameters. Circularization of the HGAP contigs was performed with Circlator (Hunt et al., 2015). In some instances (MAI134, MAI95), it was necessary to iteratively trim the ends of the initial HGAP contig by 2 kb increments until Circlator successfully circularize the sequence. For strain MAI99, circularization was achieved with the minimus2 pipeline^1^. The absence of obvious structural anomaly was manually verified by inspecting the coverage plot of mapped reads on the SMRT View browser. Genome sequence finishing included several rounds of Quiver polishing (RS_Resequencing protocol) until the count of variant ceased to decrease in additional polishing rounds.

Polished sequenced were submitted to GenBank under the accession numbers indicated in **Table 1** and automatically annotated with the NCBI Prokaryotic Genome Annotation Pipeline. The displayed multiple- whole genome alignment was generated with progressiveMauve and visualized using Mauve 2.4.0 (Darling et al., 2010).

### Phylogenetic Analysis

In addition to the Malian genome sequences determined in this study, the following reference genomes (with GB accession or Bioproject ID) where obtained from GenBank: *Xoo* MAI1 (PRJNA427174), *Xoo* CFBP1947 (NZ_CP013666), *Xoo*, BAI3 (PRJNA427174), *Xoo* NAI8 (NZ_AYSX01000001.1), *Xoo* PXO99A (NC_010717.2), *Xoo* PXO86 (NZ_CP007166.1), *Xoo* KACC10331 (AE013598.1), *Xoc* BLS256 (NC_017267.2), *Xoc* RS105 (NZ_CP011961.1), *Xoc* CFBP7341 (NZ_CP011959.1), *Xoc* CFBP7342 (NZ_CP007221.1), *X. campestris* NCPPB4346 (NZ_LHUK01000001.1), *X. oryzae* X11-5A (LHUJ01000001.1). The core genome SNPs matrix for strains under analysis was generated with the aligner module parsnp of the Harvest suite v1.1.2 (Treangen et al., 2014) enabling the -x flag to filter out SNPs in regions of recombination. Phylogeny reconstruction was conducted with RAxML version 8.2.9 (Stamatakis, 2014) using the following relevant parameters: ‘-V -T 5 -f -N 1000 -m ASC_GTRCATX –asc-corr = lewis.’ For tree inference, RAxML was run in the rapid Bootstrap analysis and search for best-scoring ML tree mode with 1,000 bootstrap replicates and SNP ascertainment bias correction. The GTR model of nucleotide substitution without rate heterogeneity and invariant site was selected based on preliminary tests with jModeltest 2.1 (Darriba et al., 2012). Trees manipulation and display were performed using the R packages ape (Paradis et al., 2004) and ggtree (Yu et al., 2017).

### TALE Analysis

Genomes were scanned for presence of TALE coding sequences. This was made using both a hidden a hidden Markov model built from sequences from TALE (Finn et al., 2015) as well as blast (Altschul et al., 1997). CRRs and RVD sequences were extracted using an in-house perl script that identified the first seven amino-acids for each repeat based on known repeat sequences as included in the QueTAL suite (Pérez-Quintero et al., 2015).

Alignments of coded TALE sequences were made using the program Arlem (Abouelhoda et al., 2010), as implemented in DisTAL as previously described (Pérez-Quintero et al., 2015), the program was modified to output alignment scores normalized by alignment length. DisTAL was also modified to output groups of TALEs and multiple alignments for each group. The modified version can be found at https://sourceforge.net/projects/tal-evolution-scripts/ and will be added to the QueTAL suite. Groups of TAL effectors were defined by cutting a DisTAL tree at a height equivalent to a score of 4.8 (roughly equivalent to 75% of the alignment consisting of highly similar repeats).

Heatmaps and alignment visualizations were created using the complexHeamap package^2^. For alignments showing color-coded repeat as those in **Figures 5**, **6B**, a vector of colors was generated based on positions and distances of unique repeats in a NJ tree, for this, the tree was cut at a height equivalent to 95% amino-acid identity, and unique colors were assigned to each resulting subgroups of repeats. Colors were assigned based on the position of each subgroup in a tree using the hue_pal function of the scales package^3^. All other figures were generated using the ggplot2 package^4^

## Author Contributions

HD, GR, FA, and ET performed the experiments. HD, AP-Q, GR, BS, RK, and SC analyzed the data. CT contributed materials. HD, OK, VV, and SC planned and designed the research. HD and SC wrote an initial version of the manuscript that was subsequently critically revised by all authors.

## Conflict of Interest Statement

The authors declare that the research was conducted in the absence of any commercial or financial relationships that could be construed as a potential conflict of interest.

## Footnotes

1 https://github.com/PacificBiosciences/Bioinformatics-Training/wiki/Circularizing-and-trimming

2 https://bioconductor.org/packages/release/bioc/html/ComplexHeatmap.html

3 https://cran.r-project.org/web/packages/scales/index.html

4 https://cran.r-project.org/web/packages/ggplot2/index.html

## Supplementary Material

FIGURE S1 Scatter plot of the virulence of Malian strains on *O. glaberrima* cv. CG14 bearing *xa41(t)* versus the susceptible reference line Azucena. Each strain is represented by a colored point whose position reflects mean lesion length obtained 14 days after leaf clipping inoculation of CG14 (*y*-axis) or Azucena (*x*-axis). Vertical and horizontal error bars correspond to the standard error of the mean on CG14 and Azucena, respectively. The color of the dots matches the year of isolation of the strain (see color key on the plot). This graph aggregates data obtained in the course of four independent experiments. Each strain has been tested in at least two independent experiment and the statistics were computed based on at least three replicate measurements.

FIGURE S2 The *talC* gene contains a 69 nucleotides deletion in the coding sequence of the N-terminal domain. Alignment of MAI1 *tal* coding sequences. Numbers on the right refer to positions relative the fist nucleotide of the initiation codon. The binding sites for the primers used for PCR amplification are depicted by underlined regions of the *talC* sequence.

FIGURE S3 Pairwise counts of polymorphic SNPs across *X. oryzae* genomes included in the phylogenetic analysis. Counts were obtained by applying the dist.dna function from the ape package with the value ‘N’ to the model parameter to pairs of genomes in the multiple SNP alignment file generated by parsnp and that included 129,898 SNPs. Rows and columns are in the same order than tips in the RAxML tree of **Figure 4**.

FIGURE S4 Whole genomes alignment of African *Xoo* strains. Snapshot of the display generated by Mauve.

FIGURE S5 Graphical view of the location of *tal* genes in sequenced genomes of Malian *Xoo* strains.

FIGURE S6 Diversity of African *Xoo* strains TALE repeat units sequence variants found in each TALE group. Within each TALE group (columns) and across strains (rows), individual cell colors code for distinct repeat units sequence variants as defined by DisTAL. The number following the pound sign in the label of the TALE groups designates the total number of unique variants found for this group in this genome set.

FIGURE S7 DisTALE alignments of African TALE groups variants. Each multiple-alignment corresponds to a TALE group. On the left, repeat sequences are aligned using the formalism of **Figure 5**. On the right the same alignments are colored and labeled based on strings of DisTALE unique repeat ID numbers.

FIGURE S8 Fractions of shared predicted targets between TALEs of the same group. The 99 RVD sequences of African TALEs were used to predict the best 500 target EBEs with Talvez 3.1 in the promoterome of Nipponbare (sequences on both strands of the 500 bp upstream the annotated start codons of the MSU7 annotation). The resulting table was used to systematically compute the percentage of shared predicted targets between TALEs of the same group. This ratio was obtained by dividing the cardinality of the intersection of the set of unique gene targets predicted for the TALE variant in row and the set of unique gene targets predicted for the TALE variant in column by the cardinality of the set of unique gene targets predicted for the TALE variant in row. Note that the resulting matrix is not symmetric because some TALEs have several EBEs predicted on the promoter of the same gene.

FIGURE S9 Talvez prediction results of selected TalB and TalF variants for previously documented targets of members of these TALE groups. **(A)** Network representation of predictions. Edge thickness and color encode Talvez prediction score values. Edges are labeled with the rank of the rice gene in Talvez predictions for the corresponding TALE variant. **(B)** Table representation of the corresponding Talvez predictions.

TABLE S1 Positions of *tal* gene coding sequences in the genomes of African *Xoo* strains.
