# Supplementary figures and images for: Functional and Genome Sequence-Driven Characterization of tal Effector Gene Repertoires Reveals Novel Variants With Altered Specificities in Closely Related Malian Xanthomonas oryzae pv. oryzae Strains
Source: Front Microbiol. 2018 Aug 6;9:1657. doi: 10.3389/fmicb.2018.01657 (PMC6088199; doi:10.3389/fmicb.2018.01657)

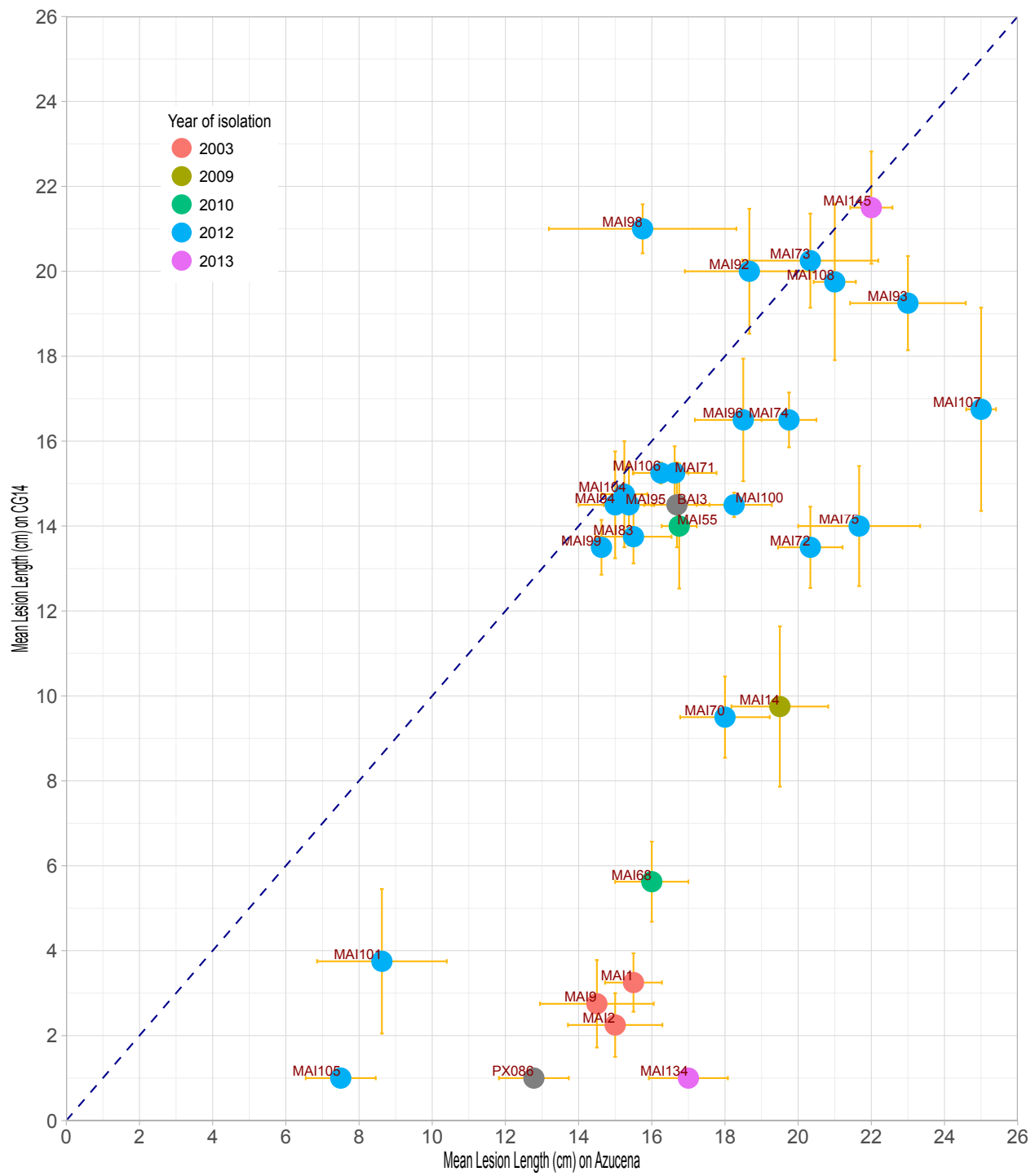

Supplement: FIGURE S1 — Scatter plot of the virulence of Malian strains on O. glaberrima cv. CG14 bearing xa41(t) versus the susceptible reference line Azucena. Each strain is represented by a colored point whose position reflects mean lesion length obtained 14 days after leaf clipping inoculation of CG14 (y-axis) or Azucena (x-axis). Vertical and horizontal error bars correspond to the standard error of the mean on CG14 and Azucena, respectively. The color of the dots matches the year of isolation of the strain (see color key on the plot). This graph aggregates data obtained in the course of four independent experiments. Each strain has been tested in at least two independent experiment and the statistics were computed based on at least three replicate measurements. [file Image_1.PDF]

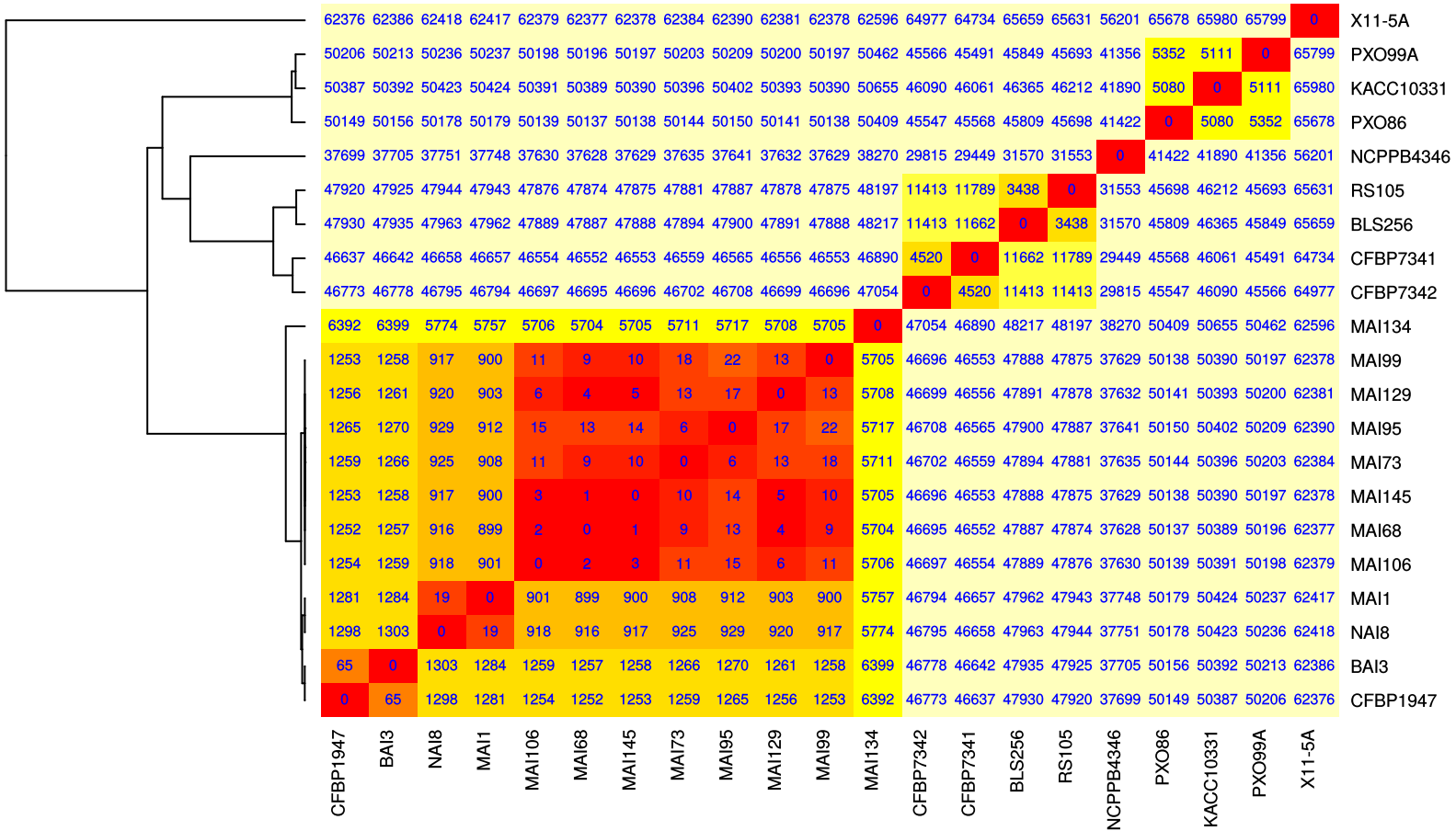

Supplement: FIGURE S3 — Pairwise counts of polymorphic SNPs across X. oryzae genomes included in the phylogenetic analysis. Counts were obtained by applying the dist.dna function from the ape package with the value ‘N’ to the model parameter to pairs of genomes in the multiple SNP alignment file generated by parsnp and that included 129,898 SNPs. Rows and columns are in the same order than tips in the RAxML tree of Figure 4. [file Image_3.PDF]

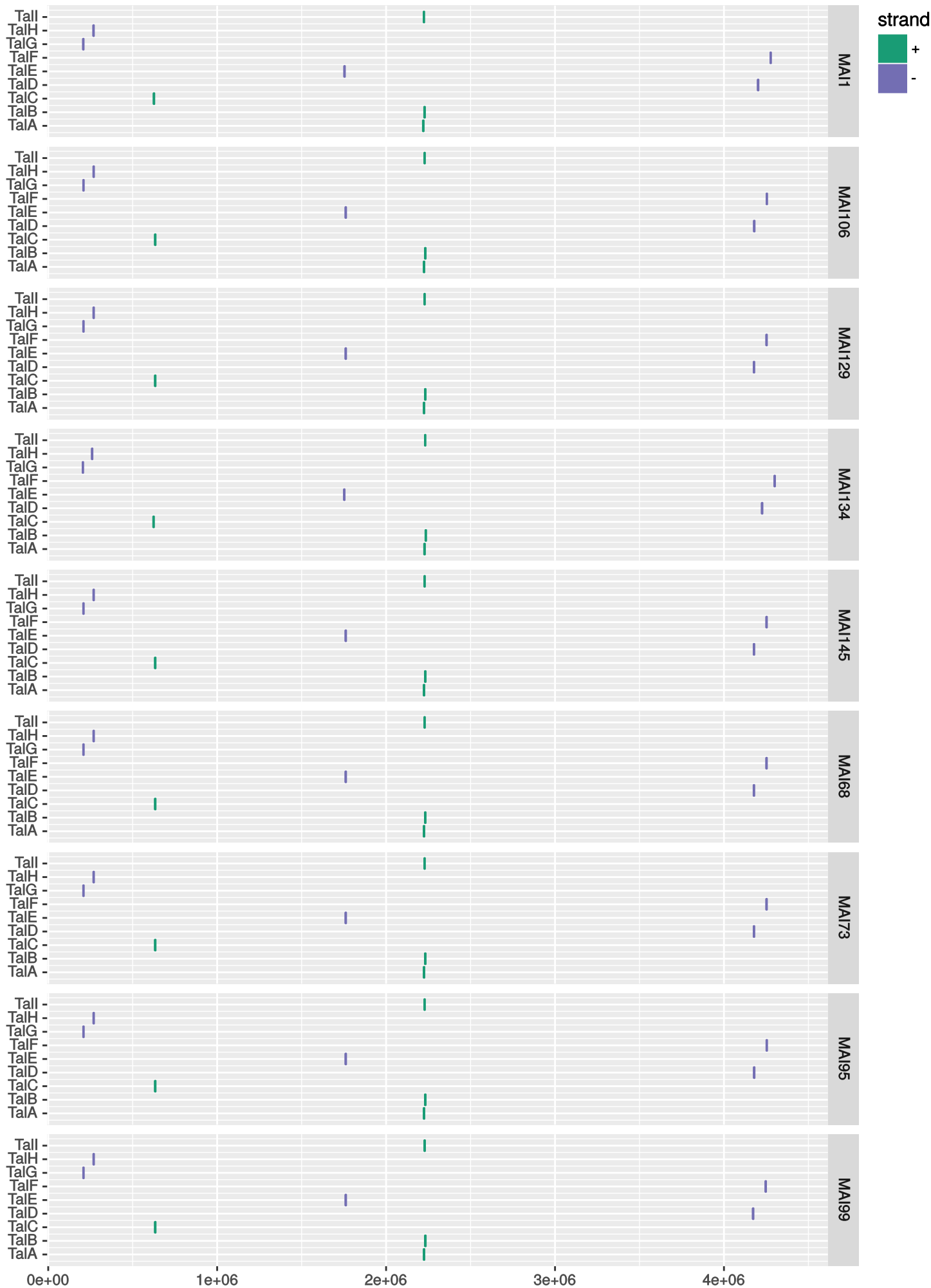

Supplement: FIGURE S5 — Graphical view of the location of tal genes in sequenced genomes of Malian Xoo strains. [file Image_5.PDF]

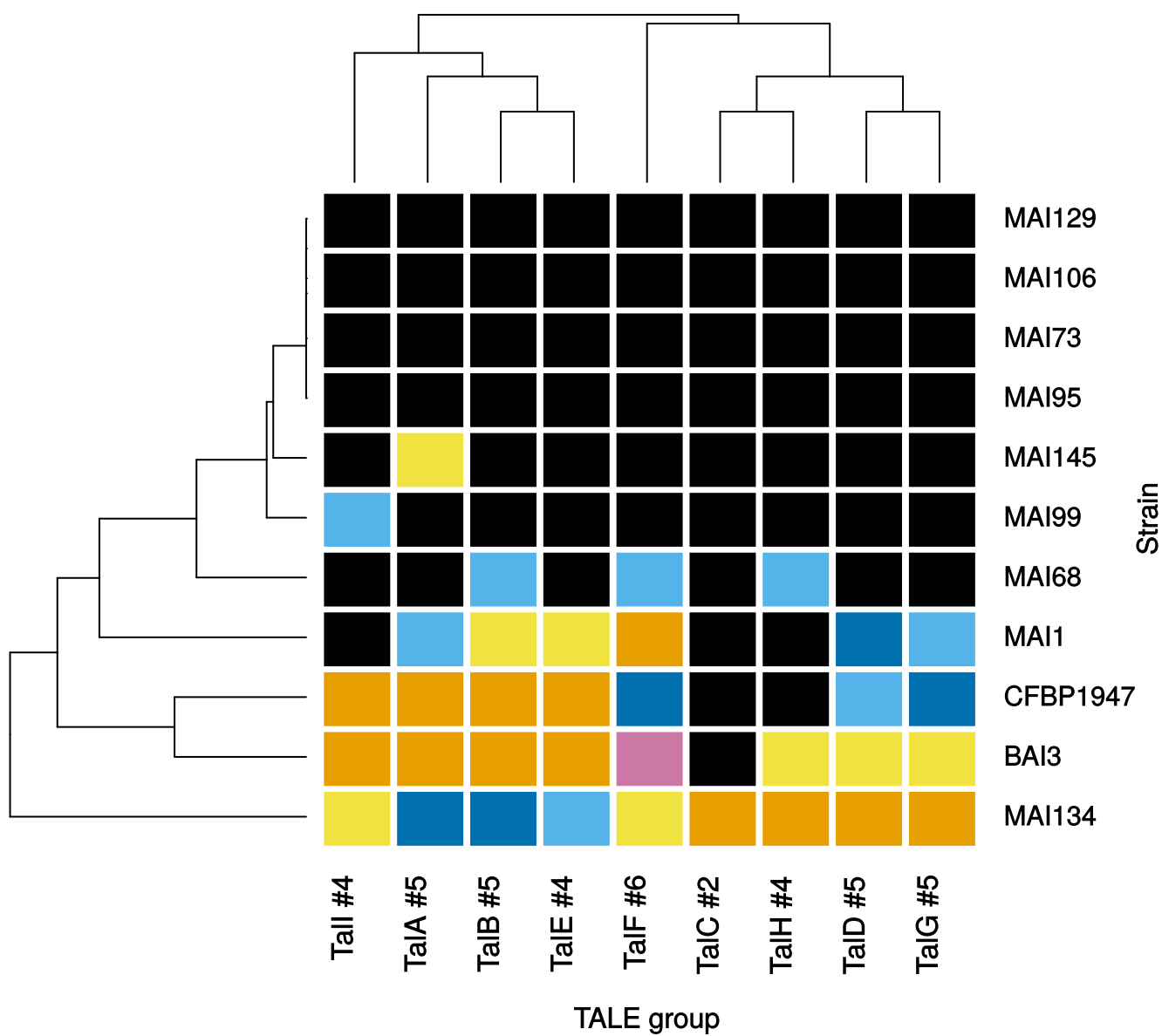

Supplement: FIGURE S6 — Diversity of African Xoo strains TALE repeat units sequence variants found in each TALE group. Within each TALE group (columns) and across strains (rows), individual cell colors code for distinct repeat units sequence variants as defined by DisTAL. The number following the pound sign in the label of the TALE groups designates the total number of unique variants found for this group in this genome set. [file Image_6.PDF]

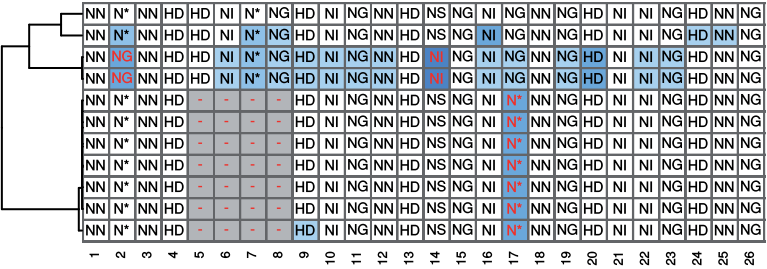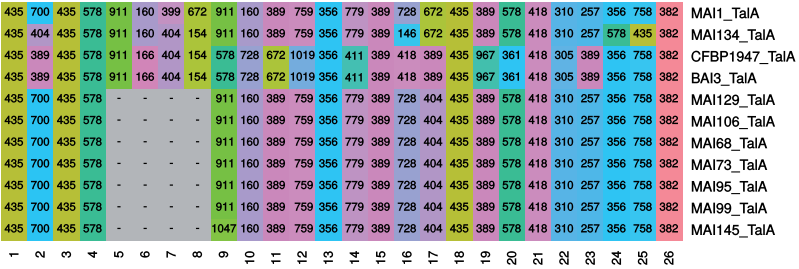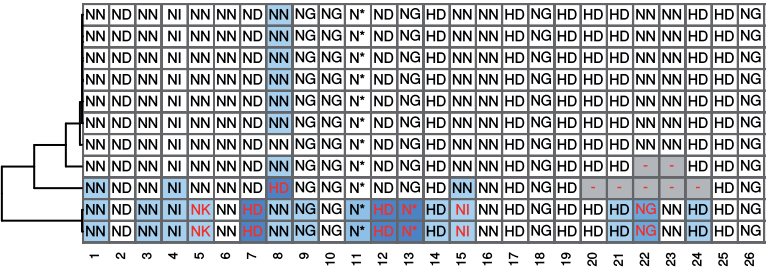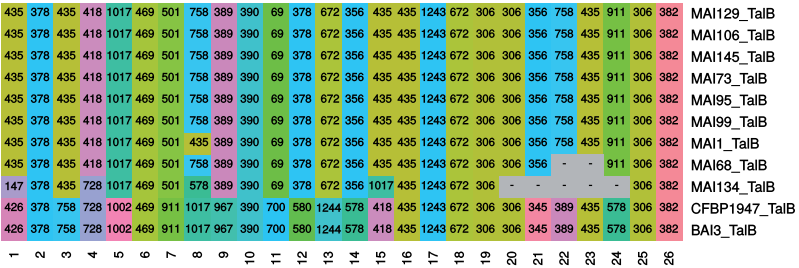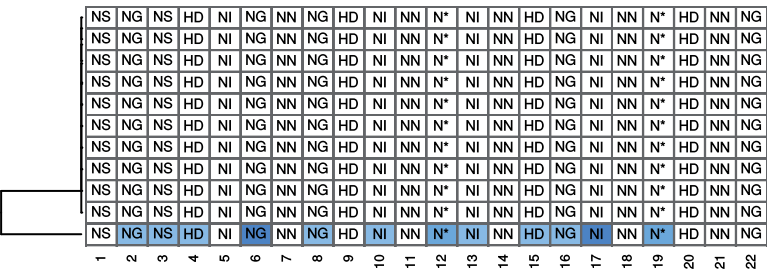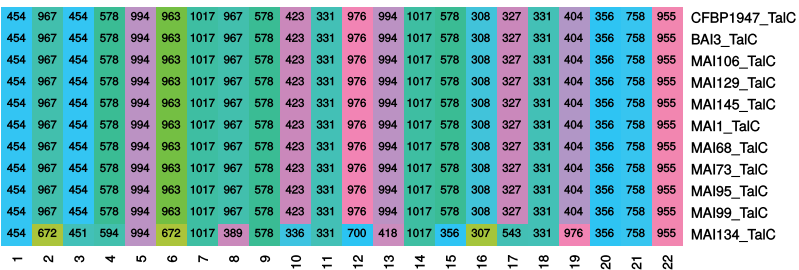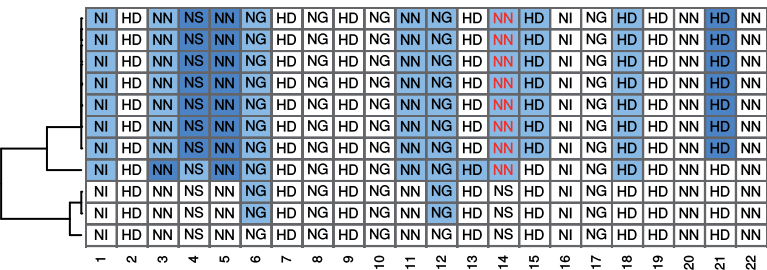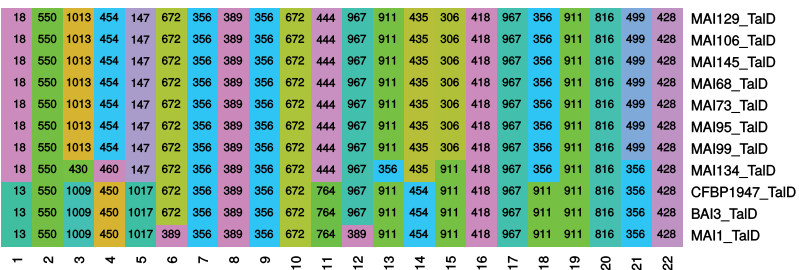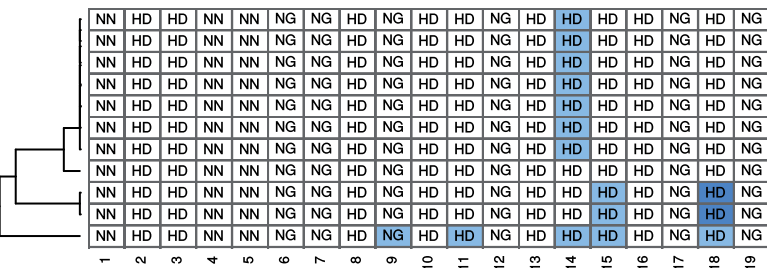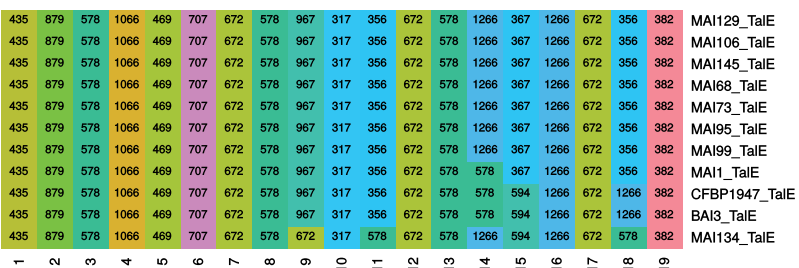

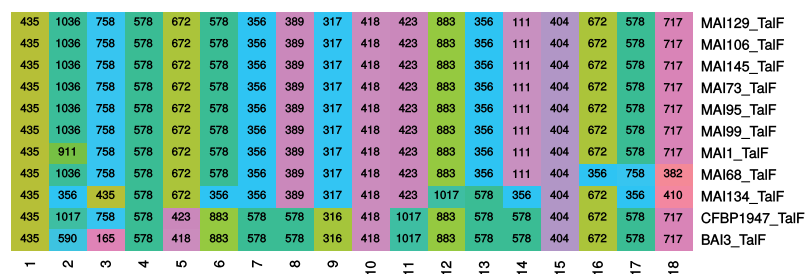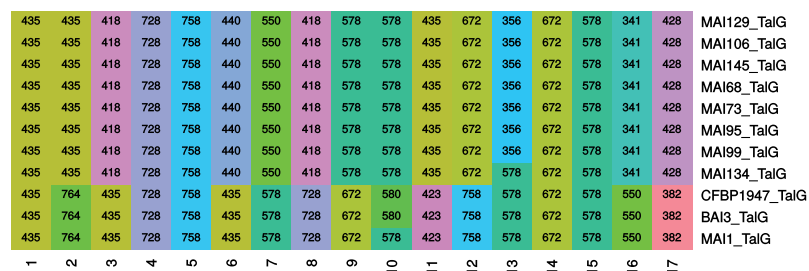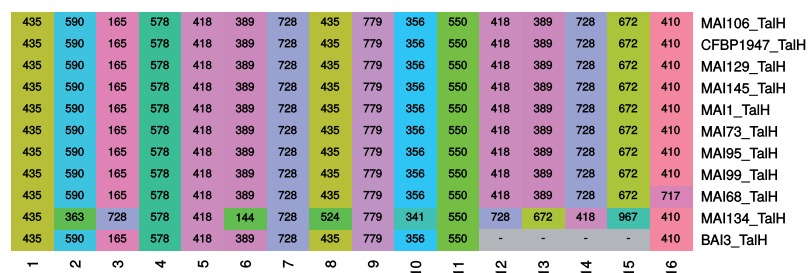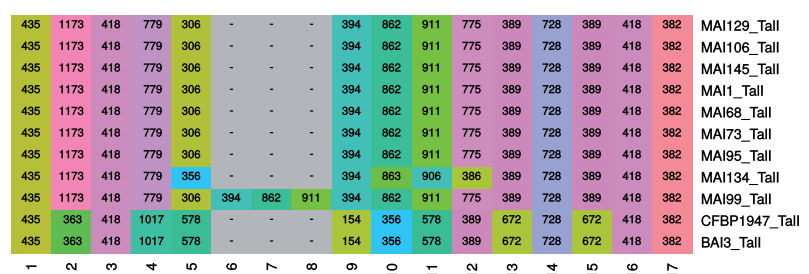

Supplement: FIGURE S7 — DisTALE alignments of African TALE groups variants. Each multiple-alignment corresponds to a TALE group. On the left, repeat sequences are aligned using the formalism of Figure 5. On the right the same alignments are colored and labeled based on strings of DisTALE unique repeat ID numbers. [file Image_7.PDF]

TaIA group

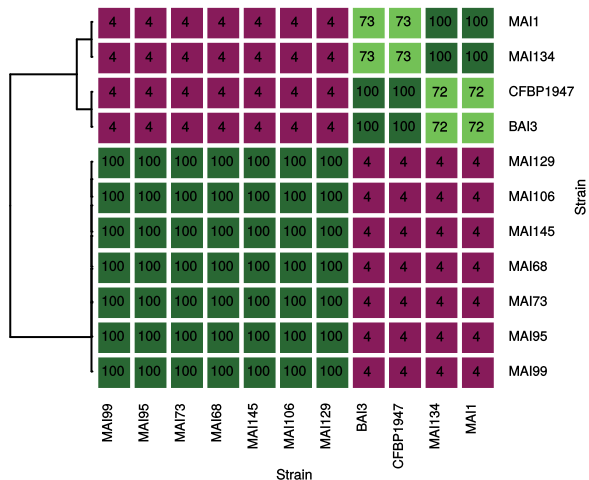

TaIB group

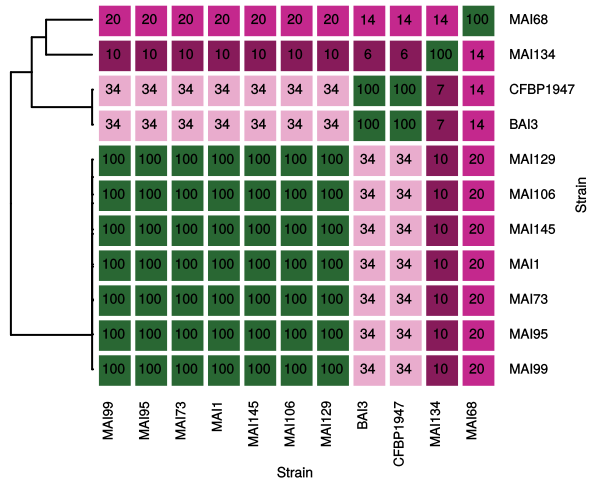

TaID group

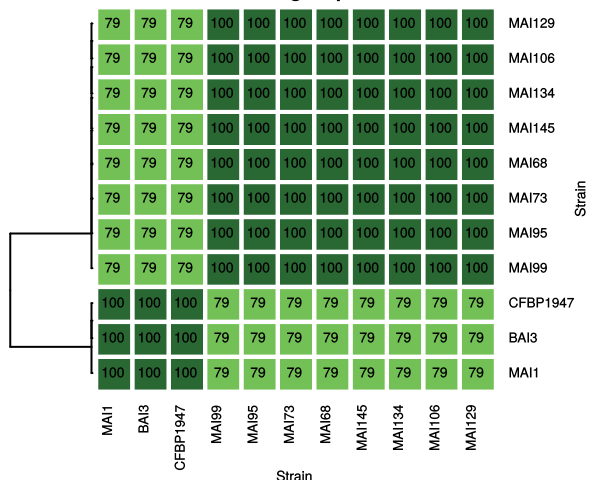

TaIF group

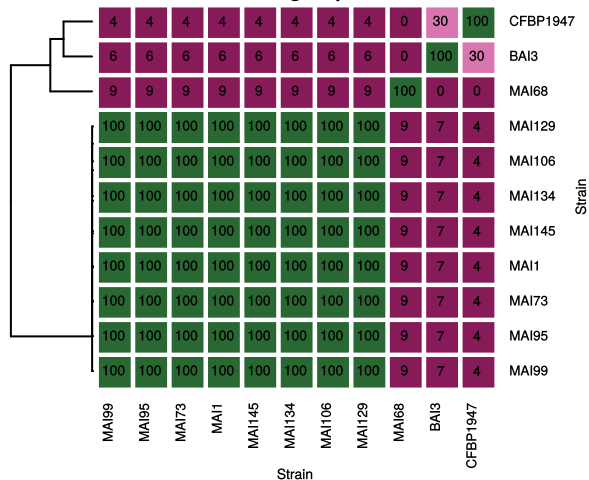

TaIG group

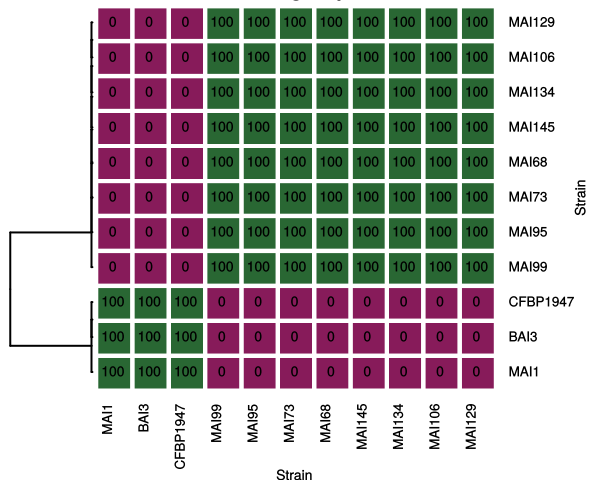

TaIH group

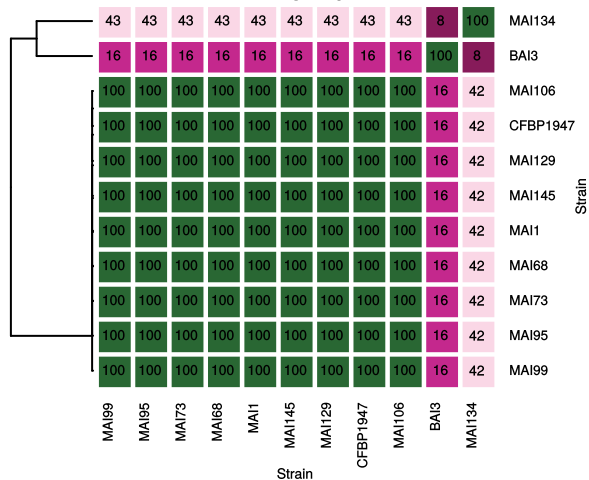

TaII group

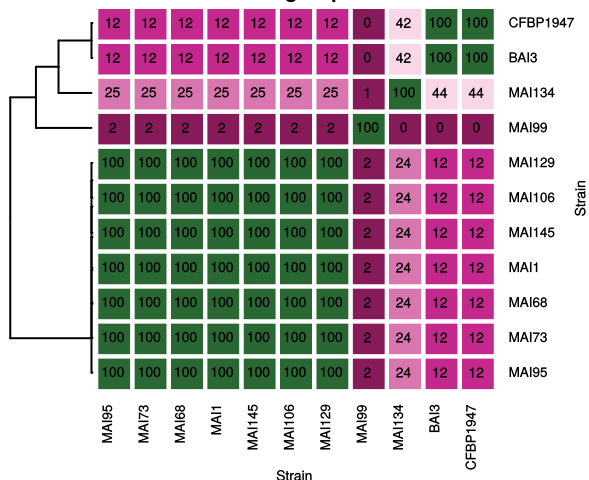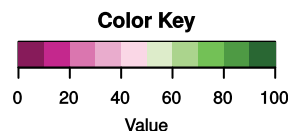

Supplement: FIGURE S8 — Fractions of shared predicted targets between TALEs of the same group. The 99 RVD sequences of African TALEs were used to predict the best 500 target EBEs with Talvez 3.1 in the promoterome of Nipponbare (sequences on both strands of the 500 bp upstream the annotated start codons of the MSU7 annotation). The resulting table was used to systematically compute the percentage of shared predicted targets between TALEs of the same group. This ratio was obtained by dividing the cardinality of the intersection of the set of unique gene targets predicted for the TALE variant in row and the set of unique gene targets predicted for the TALE variant in column by the cardinality of the set of unique gene targets predicted for the TALE variant in row. Note that the resulting matrix is not symmetric because some TALEs have several EBEs predicted on the promoter of the same gene. [file Image_8.PDF]
