# Supplementary material for: Functional and Genome Sequence-Driven Characterization of tal Effector Gene Repertoires Reveals Novel Variants With Altered Specificities in Closely Related Malian Xanthomonas oryzae pv. oryzae Strains
Source: Front Microbiol. 2018 Aug 6;9:1657. doi: 10.3389/fmicb.2018.01657 (PMC6088199; doi:10.3389/fmicb.2018.01657)
Supplement: FIGURE S9 — Talvez prediction results of selected TalB and TalF variants for previously documented targets of members of these TALE groups. (A) Network representation of predictions. Edge thickness and color encode Talvez prediction score values. Edges are labeled with the rank of the rice gene in Talvez predictions for the corresponding TALE variant. (B) Table representation of the corresponding Talvez predictions. [file Image_9.PDF]

A

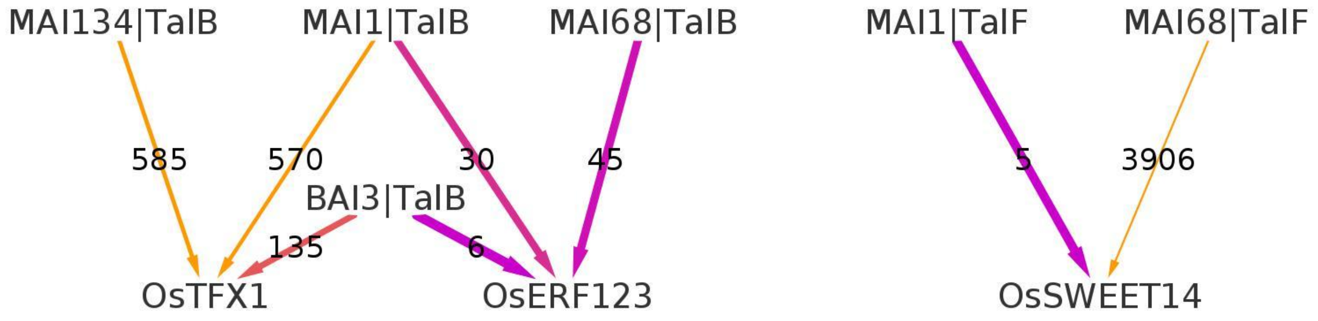

B

TALE_ID	TALE_Group	strain	target_LOCID	target_gene_name	score	rank	EBE_seq
MAI1|TalB	TalB	MAI1	LOC_Os09g29820	OsTFX1	10,576	570	TAAAAGGCCCTCACCAACCCATCGCCT
BAI3|TalB	TalB	BAI3	LOC_Os09g29820	OsTFX1	12,716	135	TAAAAGGCCCTCACCAACCCATCGCCT
MAI134|TalB	TalB	MAI134	LOC_Os09g29820	OsTFX1	10,891	585	TAAAAGGCCCTCACCAACCCAT
MAI1|TalB	TalB	MAI1	LOC_Os09g39810	OsERF123	13,769	30	TGCGATGCGTTTCCCACCTCCCACCTC
MAI68|TalB	TalB	MAI68	LOC_Os09g39810	OsERF123	14,499	45	TGCGATGCGTTTCCCACCTCCCACC
BAI3|TalB	TalB	BAI3	LOC_Os09g39810	OsERF123	15,909	6	TGCGATGCGTTTCCCACCTCCCACCTC
MAI1|TalF	TalF	MAI1	LOC_Os11g31190	OsSWEET14	15,146	5	TAAGCTCATCAAGCCTTCA
MAI68|TalF	TalF	MAI68	LOC_Os11g31190	OsSWEET14	8,843	3906	TAAGCTCATCAAGCCTTCA
