# Supplementary material for: Functional and Genome Sequence-Driven Characterization of tal Effector Gene Repertoires Reveals Novel Variants With Altered Specificities in Closely Related Malian Xanthomonas oryzae pv. oryzae Strains
Source: Front Microbiol. 2018 Aug 6;9:1657. doi: 10.3389/fmicb.2018.01657 (PMC6088199; doi:10.3389/fmicb.2018.01657)
Supplement: FIGURE S2 — The talC gene contains a 69 nucleotides deletion in the coding sequence of the N-terminal domain. Alignment of MAI1 tal coding sequences. Numbers on the right refer to positions relative the fist nucleotide of the initiation codon. The binding sites for the primers used for PCR amplification are depicted by underlined regions of the talC sequence. [file Image_2.PDF]

```
MAI1_TalC  GCCGTCGGCACGCCTCATACAGAGGCTGCCCCAGCAGAGGGGGATGAGGTGCAATCGGCTCTGCGTGCAGCCGATGACCCGCCACCCACC 360
MAI1_TalA  GCCGTCGGCACGCCTCATACAGAGGTTGCCCCAGCAGAGGGGGATGAGGTGCAATCGGCTCTGCGTGCAGCCGATGACCCGCCACCCACC 360
MAI1_TalI  GCCGTCGGCACGCCTCATACAGAGGCTGCCCCAGCAGAGGGGGATGAGGTGCAATCGGCTCTGCGTGCAGCCGATGACCCGCCACCCACC 360
MAI1_TalB  GCCGTCGGCACGCCTCATACAGAGGCTGCCCCAGCAGAGGGGGATGAGGTGCAATCGGCTCTGCGTGCAGCCGATGACCCGCCACCTACC 360
MAI1_TalF  GCCGTCGGCACGCCTCATACAGAGGCTGCCCCAGCAGAGGGGGATGAGGTGCAATCGGCTCTGCGTGCAGCCGATGACCCGCCACCCACC 360
MAI1_TalD  GCCGTCGGCACGCCTCATACAGAGGCTGCCCCAGCAGAGGGGGATGAGGTGCAATCGGCTCTGCGTGCAGCCGATGACCCGCCACCCACC 360
MAI1_TalE  GCCGTCGGCACGCCTCATACAGAGGCTGCCCCAGCAGAGGGGGATGAGGTGCAATCGGCTCTGCGTGCAGCCGATGACCCGCCACCCACC 360
MAI1_TalH  GCCGTCGGCACGCCTCATACAGAGGCTGCCCCAGCAGAGGGGGATGAGGTGCAATCGGCTCTGCGTGCAGCCGATGACCCGCCACCCACC 360
MAI1_TalG  GCCGTCGGCACGCCTCATACAGAGGCTGCCCCAGCAGAGGGGGATGAGGTGCAATCGGCTCTGCGTGCAGCCGATGACCCGCCACCCACC 360

MAI1_TalC  GTGCGTGTCGCTGTCACTGC---------------------------------------------------------------------C 381
MAI1_TalA  GTGCGTGTCGCTGTCACTGCCGCGCGGCAGCCGCGCGCCAAGCCGGCCCCGCGA---CGTGCTGCGCAACCCTCCGACGCTTCGCCGGCC 447
MAI1_TalI  GTGCGTGTCGCTGTCACTGCCGCGCGGCAGCCGCGCGCCAAGCCGGCCCCGCGACGGCGTGCTGCGCAACCCTCCGACGCTTCGCCGGCC 450
MAI1_TalB  GTGCGTGTCGCTGTCACTGCCGCGCGGCAGCCGCGCGCCAAGCCGGCCCCGCGA---CGTGCTGCGCAACCCTCCGACGCTTCGCCGGCC 447
MAI1_TalF  GTGCGTGTCGCTGTCACTGCCGCGCGGCAGCCGCGCGCCAAGCCGGCCCCGCGACGGCGTGCTGCGCAACCCTCCGACGCTTCGCCGGCC 450
MAI1_TalD  GTGCGTGTCGCTGTCACTGCCGCGCGGCAGCCGCGCGCCAAGCCGGCCCCGCGACGGCGTGCTGCGCAACCCTCCGACGCTTCGCCGGCC 450
MAI1_TalE  GTGCGTGTCGCTGTCACTGCCGCGCGGCAGCCGCGCGCCAAGCCGGCCCCGCGA---CGTGCTGCGCAACCCTCCGACGCTTCGCCGGCC 447
MAI1_TalH  GTGCGTGTCGCTGTCACTGCCGCGCGGCAGCCGCGCGCCAAGCCGGCCCCGCGA---CGTGCTGCGCAACCCTCCGACGCTTCGCCGGAC 447
MAI1_TalG  GTGCGTGTCGCTGTCACTGCCGCGCGGCAGCCGCGCGCCAAGCCGGCCCCGCGACGGCGTGCTGCGCAACCCTCCGACGCTTCGCCGGCC 450

MAI1_TalC  GCGCAGGTGGATCTACGCACGCTCGGCTACAGT---CAGCAGCAAGAGAAGATCAAACCGAATGTTCGTTCGACAGTGGCGCAGCACCAC 468
MAI1_TalA  GCGCAGGTGGATCTACGCACGCTCGGCTACAGTCAGCAGCAGCAAGAGAAGATCAAACCGAATGTTCGTTTGACAGTGGCGCAGCACCAC 537
MAI1_TalI  GCGCAGGTGGATCTACGCACGCTCGGCTACAGTCAGCAGCAGCAAGAGAAGATCAAACCGAATGTTCGTTTGACAGTGGCGCAGCACCAC 540
MAI1_TalB  GCGCAGGTGGATCTACGCACGCTCGGCTACAGTCAGCAGCAGCAAGAGAAGATCAAACCGAATGTTCGTTTGACAGTGGCGCAGCACCAC 537
MAI1_TalF  GCGCAGGTGGATCTACGCACGCTCGGCTACAGTCAGCAGCAGCAAGAGAAGATCAAACCGAATGTTCGTTCGACAGTGGCGCAGCACCAC 540
MAI1_TalD  GCGCAGGTGGATCTACGCACGCTCGGCTACAGTCAGCAGCAGCAAGAGAAGATCGAACCGAATGTTCGTTCGACAGTGGCGCAGCACCAC 540
MAI1_TalE  GCGCAGGTGGATCTACGCACGCTCGGCTACAGTCAGCAGCAGCAAGAGAAGATCGAACCGAATGTTCGTTTGACAGTGGCGCAGCACCAC 537
MAI1_TalH  GCGCAGGTGGATCTACGCACGCTCGGCTACAGTCAGCAGCAGCAAGAGAAGATCGAACCGAATGTTCGTTCGACAGTGGCGCAGCACCAC 537
MAI1_TalG  GCGCAGGTGGATCTACGCACGCTCGGCTACAGTCAGCAGCAGCAAGAGAAGATCGAACCGAATGTTCGTTCGACAGTGGCGCAGCACCAC 540

MAI1_TalC  GAGGCACTGGTGGGCCATGGGTTTACACACGCGCACATCGTTGCGCTCAGCCGACACCCGGCAGCGTTAGGGACCGTCGCTGTCAAGTAT 558
MAI1_TalA  GAGGCACTGGTGGGCCATGGGTTTACACACGCGCACATCGTTGCGCTCAGCCGACACCCGGCAGCGTTAGGGACCGTCGCTGTCAAGTAT 627
MAI1_TalI  GAGGCACTGGTGGGCCATGGGTTTACACACGCGCACATCGTTGCGCTCAGCCAACACCCGGCAGCGTTAGGGACCGTCGCTGTCAAGTAT 630
MAI1_TalB  GAGGCACTGGTGGGCCATGGGTTTACACACGCGCACATCGTTGCGCTCAGCCAACACCCGGCAGCGTTAGGGACCGTCGCTGTCAAGTAT 627
MAI1_TalF  GAGGCACTGGTGGGCCATGGGTTTACACACGCGCACATCGTTGCGCTCAGCCGACACCCGGCAGCGTTAGGGACCGTCGCTGTCAAGTAT 630
MAI1_TalD  GAGGCACTGGTGGGCCATGGGTTTACACACGAGCACATCGTTGCGCTCAGCCGACACCCGGCAGCGTTAGGGACCGTCGCTGTCAAGTAT 630
MAI1_TalE  GAGGCACTGGTGGGCCATGGGTTTACACACGCGCACATCGTTGCGCTCAGCCAACACCCGGCAGCGTTAGGGACCGTCGCTGTCAAGTAT 627
MAI1_TalH  GAGGCACTGGTGGGCCATGGGTTTACACACGCGCACATCGTTGCGCTCAGCCGACACCCGGCAGCGTTAGGGACCGTCGCTGTCAAGTAT 627
MAI1_TalG  GAGGCACTGGTGGGCCATGGGTTTACACACGCGCACATCGTTGCGCTCAGCCGACACCCGGCAGCGTTAGGGACCGTCGCTGTCAAGTAT 630
```
